# Characterization of Tyrosinase Inhibitors in *Dryopteris crassirhizoma* Rhizome Using a Combination of High-Speed Counter-Current Chromatography, Affinity-Based Ultrafiltration, and Liquid Chromatography–Tandem Mass Spectrometry

**DOI:** 10.3389/fnut.2022.862773

**Published:** 2022-04-18

**Authors:** Zhiqiang Wang, Ning Wang, Dandan Han, Hongyuan Yan

**Affiliations:** ^1^Key Laboratory of Public Health Safety of Hebei Province, School of Public Health, Hebei University, Baoding, China; ^2^Key Laboratory of Medicinal Chemistry and Molecular Diagnosis of Ministry of Education, College of Pharmaceutical Sciences, Hebei University, Baoding, China

**Keywords:** tyrosinase, *Dryopteris crassirhizoma*, affinity-based ultrafiltration, melanin content, zebrafish

## Abstract

Dryopteris crassirhizoma rhizome (DCR) inhibits melanin production in B16F10 melanoma cells and tyrosinase activity. The melanin content and tyrosinase activity of DCR-treated zebrafish embryos were determined to evaluate the *in vivo* inhibitory effect of DCR on melanogenesis. Moreover, an off-line hyphenated method combining the high-speed counter-current chromatography, affinity-based ultrafiltration, and liquid chromatography–tandem mass spectrometry was used to identify and characterize the DCR compounds with tyrosinase inhibitory activity. Our results indicated that DCR significantly decreased the melanin content and tyrosinase activity in zebrafish embryos in a dose-dependent manner; moreover, 22 compounds in DCR presented tyrosinase inhibitory activity. *In silico* molecular docking prediction data indicated that the 22 compounds in DCR can form stable conformations in the active site pocket of tyrosinase.

## Introduction

Melanin is a naturally occurring skin pigment that protects the human body against ultraviolet (UV) radiation damage (1). Recently, melanin biosynthesis has attracted considerable attention because of the increasing occurrence of melanomas, freckles, chloasma, and senile spots caused by skin hyperpigmentation (2–4). Tyrosinase, which is the primary monooxygenase during melanin synthesis, converts tyrosine into dopquinone *via* 3-dihydroxyphenylalanine as an intermediate through a two-step catalytic process (5, 6). Therefore, numerous tyrosinase inhibitors have been developed to decrease the melanin synthesis rate and diminish hyperpigmentation. Nevertheless, the weak *in vivo* activity of tyrosinase inhibitors and safety concerns associated with them have hindered their practical use (7). Recently, the use of functional foods has gained popularity as consumers are paying an increasing attention to diet and health. Therefore, the development of safe functional foods that can effectively inhibit tyrosinase activity and melanogenesis has attracted considerable attention.

Pteridophyta plants, commonly known as ferns, have historically provided many health benefits to humans and have been used as food, teas, and herbal medicines by the Chinese, Indians, and Native Americans since ancient times. However, even though these plants are appreciated for their esthetic and medicinal properties, their potential applications and economic value are still underestimated (8). The rhizome of *Dryopteris crassirhizoma* Nakai, which is a perennial herbaceous fern species belonging to the *Dryopteridaceae* family, has been used to treat viral diseases (9, 10). Previous studies have demonstrated that *D. crassirhizoma* rhizome (DCR) significantly inhibited melanin production in B16F10 melanoma cells and also inhibited tyrosinase activity (11, 12). These findings suggested that DCR is a natural tyrosinase inhibitor that can be used as a functional food ingredient. However, the inhibitory effect of DCR on melanogenesis *in vivo* has not yet been investigated. Moreover, the DCR compounds with tyrosinase inhibitory activity should be identified and characterized.

Owing to the complex chemical composition of natural product extracts and high structural diversity of their components, the identification of biologically active compounds using bioassay-guided isolation is laborious (13). Recently, affinity-based ultrafiltration hyphenated with liquid chromatography–tandem mass spectrometry (LC–MS/MS) has been used for the *in situ* identification of bioactive compounds in natural product extracts (14, 15). However, the detection of the minor bioactive compounds captured by proteins *via* affinity-based ultrafiltration using LC–MS/MS is challenging because the significant differences in extracts composition cause the signals of the major components to mask those of the minor components (16). High-speed countercurrent chromatography (HSCCC) is a separation method that uses liquid–liquid partition without a solid phase. HSCCC does not entail irreversible adsorption. Therefore, despite the similar polarities of the minor and major components, the minor components of extracts can be enriched from the major components without loss using the differences in partition coefficients (17, 18). Consequently, the combination of HSCCC, affinity-based ultrafiltration, and LC–MS/MS can be a promising method for the identification of bioactive compounds from natural products.

Therefore, in this study, the melanin content and tyrosinase activity of zebrafish embryos were investigated to assess the effect of DCR on melanogenesis *in vivo*. Moreover, the tyrosinase inhibitors in DCR were identified and characterized using a combination of HSCCC, ultrafiltration, and LC–MS/MS.

## Materials and Methods

### Reagents

Arbutin, dimethyl sulfoxide, L-tyrosine, and mushroom tyrosinase were provided by Sigma-Aldrich (St. Louis, MO, United States). Tricaine, embryo culture medium, methylcellulose, and lytic buffer were supplied by Shandong Yixiyue Biotechnology Co., Ltd. (Weifang, China). Phosphate-buffered saline (PBS; pH 7.2) was obtained from Gibco (Waltham, MA, United States). Dipotassium hydrogen phosphate, potassium dihydrogen phosphate, and NaOH were acquired from Aladdin (Shanghai, China). All the organic solvents were supplied by Concord Technology (Tianjin, China). A Milli-Q water purification system (Millipore, Billerica, MA, United States) was used to obtain ultrapure water (18.2 MΩ cm), which was used for all the experiments.

### Preparation of the *Dryopteris crassirhizoma* Rhizome Extract

*Dryopteris crassirhizoma* rhizome (voucher WLL-2018-03) was provided by Yiyuan Chinese Herb Medicine Co., Ltd. (Anguo, Hebei, China) in August 2018 and stored at the College of Public Health, Hebei University. Dried ground DCR (10 g) was extracted in triplicate with 100 ml of a 90% methanol aqueous solution over 24 h, and 1.59 g of solid extract powder was obtained after filtration, concentration, and lyophilization.

### Tyrosinase Assay

Mixtures of DCR extract samples with different concentrations, L-tyrosine (0.3 mM), and mushroom tyrosinase (50 units/ml) were incubated in PBS at 25°C, and their absorbances were recorded at 450 nm after 30 min. Arbutin, which was the positive control, was used as the reference compound. The tyrosinase inhibition values were calculated as follows:


Inhibition(%)=[1-(A-B)/(C-D)]×100%,

where A is the absorbance of the reaction system comprising the DCR extract sample and tyrosinase, B is the absorbance of the reaction system comprising only the DCR extract sample, C is the absorbance of the reaction system comprising only tyrosinase, and D is the absorbance of the reaction mixture without DCR extract samples and tyrosinase.

### Zebrafish Maintenance and Breeding

A pair of healthy broodstock zebrafish was placed in an incubator equipped with a barrier, which was used to separate the male and female zebrafish overnight prior to mating. The barrier was removed the next morning, and light was used to stimulate the broodstock zebrafish to spawn. Then, 4 h post-fertilization (hpf), the zebrafish embryos were cultured with DCR (10, 50, and 100 μg/ml in embryo culture medium), arbutin (10, 50, and 100 μg/ml in embryo culture medium), or embryo culture medium in 96-well plates at 28°C under 14/10 h light/dark cycles. Each group comprised 60 embryos, and the corresponding culture media were changed daily. A stereomicroscope was used to record the melanin changes in the zebrafish embryos 72 hpf.

### Determination of Tyrosinase Activity of Zebrafish

After treatment, 50 zebrafish embryos from each group were homogenized in the PBS using a tissue homogenizer, and the homogenates were centrifuged. The supernatant of each group was collected and used to determine the tyrosinase activity of the group. After protein concentration was normalized, the diluted supernatants containing tyrosinase (160 μl) and L-tyrosine solution (40 μl) were mixed and incubated at 37°C. After 1 h, 450 nm UV light was used to determine the absorbance of each mixture, and the tyrosinase inhibition values were calculated as follows:


$$\text{Inhibition}\left(\%\right)=\left(1-A/{\text{A}}_{0}\right)\times 100\%,$$

where A is the absorbance of L-tyrosine incubated with the supernatant obtained from the zebrafish embryos treated with DCR or arbutin and A_0_ is the absorbance of L-tyrosine incubated with the supernatant obtained from the zebrafish embryos treated with embryo culture medium.

### Assessment of Melanin Content of Zebrafish

To assess the melanin content of each group of zebrafish, the melanin precipitate from each group was collected and suspended in 400 μl NaOH (1 M). Next, 490 nm UV light was used to evaluate the dissolved melanin content, and the relative melanin content was calculated as follows:


$$\mathrm{Melanin}\,\mathrm{content}=A/{\text{A}}_{0}\times 100\%,$$

where A is the absorbance of the melanin samples obtained from the zebrafish embryos treated with DCR or arbutin and A_0_ is the absorbance of the melanin samples obtained from the zebrafish embryos treated with embryo culture medium only.

### High-Speed Countercurrent Chromatography Separation

A TBE-300C HSCCC instrument (Tauto Biotechnique Company, Shanghai, China) equipped with an Isolera FLASH purification system (Biotage, Uppsala, Sweden) was used for separation. Before separation, 300 ml of the stationary phase was pumped into the coiled column and subsequently rotated at 400 rpm. After equilibrium was reached, 20 ml of the DCR extract solution was injected, and a series of mobile phases was delivered into the coil in the tail-to-head mode. A mixture of *n*-butanol (*n*-BuOH) and water [1:10 (v/v)] was used as the stationary phase. The injected DCR extract was dissolved in a mixture of ethyl acetate (EtOAc), n-BuOH, and water [1:1:10, (v/v/v)] to achieve a concentration of 25 mg/ml. A list of mobile phases is presented in Figure 6. Chromatograms were recorded at a wavelength of 254 nm.

### Affinity-Based Ultrafiltration

A mixture of DCR extract solution (0.1 mg/ml) and tyrosinase (100 units/ml) was incubated at 37°C for 30 min and subsequently centrifuged using a Microcon YM-10 centrifugal filter unit (Millipore, Billerica, MA, United States) at 10,000 × *g* for 30 min. A control group without tyrosinase was analyzed in parallel. The ultrafiltrates were collected for subsequent LC–MS/MS analysis.

### Liquid Chromatography–Tandem Mass Spectrometry Analysis

An UltiMate 3000 high-performance liquid chromatography (HPLC) system (Thermo Fisher Scientific, Waltham, MA, United States) equipped with a Q Exactive Orbitrap mass spectrometer (Thermo Fisher Scientific, Waltham, MA, United States) was used for LC–MS/MS analysis. An Eclipse SB-C18 Rapid Resolution column (150 mm length, 4.6 mm ID, and 3.5 μm particle size; Agilent, Santa Clara, CA, United States) was used for separation. The sample’s injection volume and flow rate were 10 μl and 1 mg/ml, respectively. The elution gradient program was as follows: 0–8 min, 5–20% B; 8–25 min, 20–30% B; 25–60 min, 30–100% B; 60–67 min, 100% B; 67–70 min, 100–5% B; 70–80 min, 5% B; and the mobile phases comprised 0.1% formic acid in water (A) and methanol (B). The eluent was monitored at 254 nm. Peak identification was performed in the negative mode, and the electrospray ionization source conditions were set as follows: sheath gas flow rate: 45 arb, auxiliary gas flow rate: 15 arb, capillary temperature: 320°C, full mass resolution: 70,000, MS/MS resolution: 17,500, collision energy: 20/40/60 eV in the normalized collision energy model, and spray voltage: −3.1kV.

### *In silico* Docking

*In silico* docking was performed using the Surflex-Dock program version (Tripos, St. Louis, MO, United States), and the crystal structure of tyrosinase (2Y9X) was retrieved from the RCSB Protein Data Bank. Prior to docking, the macromolecules and small molecules were prepared, including structural file retrieval, water molecule removal, non-protein atom removal, structural defect correction, and molecular energy minimization. The “thresh” and “bloat” parameters of the docking protocol were set to be 0.5 and 1, respectively. The PyMOL (Schrödinger, New York, NY, United States) and LigPlot software (EMBL-EBI, Cambridge, United Kingdom) were used for data visualization.

### Statistical Analysis

All experiments were performed at least in triplicate, and the results are expressed as means ± standard deviations (SDs). Data analysis was performed using the SPSS software (IBM, Armonk, NY, United States). The mean values were compared using Student’s unpaired *t*-test or one-way analysis of variance (ANOVA), and statistical significance was set at *p* < 0.05.

## Results and Discussion

### Tyrosinase Inhibitory Activity of *Dryopteris crassirhizoma* Rhizome

**FIGURE 1:**
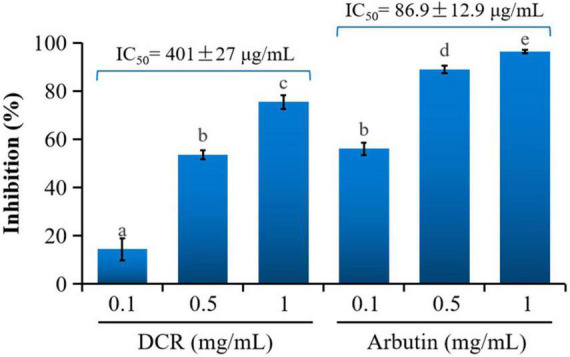
Inhibitory activity of *Dryopteris crassirhizoma* rhizome (DCR) on mushroom tyrosinase. *IC*_50_ is the concentration of the sample with a mushroom tyrosinase inhibitory activity of 50%. Different lower case letters indicate significant differences (*p* < 0.05).

The inhibitory effects of various concentrations of the DCR extracts on mushroom tyrosinase were tested *in vitro*. The DCR extracts considerably inhibited mushroom tyrosinase, and the inhibitions of the samples with DCR extract concentrations of 0.1, 0.5, and 1 mg/ml were 14.32, 53.61, and 75.47%, respectively (Figure 1). The half-maximal inhibitory concentration (*IC*_50_) of the DCR extract for tyrosinase was 401 μg/ml. Experiments were performed using arbutin as the reference compound. The inhibitions of arbutin samples with concentrations of 0.1, 0.5, and 1 mg/ml were 56.03, 88.90, and 96.45%, respectively, and the *IC*_50_ of arbutin was 86.9 μg/ml. The tyrosinase inhibitory activity of DCR was considerable yet lower than that of arbutin, as confirmed by the aforementioned experimental results and previously reported data (11, 12).

### Effect of *Dryopteris crassirhizoma* Rhizome on Melanogenesis in Zebrafish Embryos

**FIGURE 2:**
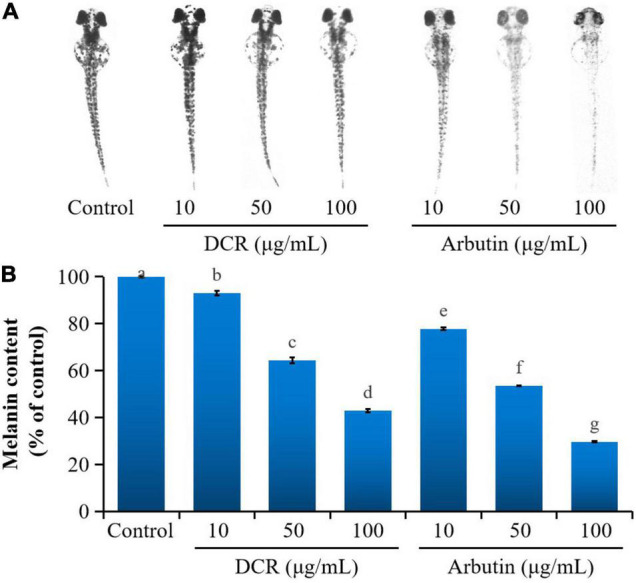
The effect of *Dryopteris crassirhizoma* rhizome (DCR) on **(A)** melanogenesis in zebrafish embryos and **(B)** melanin content of zebrafish embryos. Different lower case letters indicate significant differences (*p* < 0.05).

Mushroom tyrosinase has been commonly used to assess the tyrosinase inhibitory activity related to the melanogenesis inhibition of traditional medicinal plants and food items (19). However, the effect of DCR on melanogenesis *in vivo* remains unclear. Zebrafish embryos present significant physiological and genetic similarities with mammals. Moreover, pigmentation experiments using zebrafish require simple protocols (20). Therefore, zebrafish embryos have been increasingly used as *in vivo* test models, replacing mice and other animal models for phenotype-based melanogenesis inhibition studies. Consequently, the effect of DCR on melanogenesis inhibition was investigated using zebrafish embryos. The pigmentation of zebrafish larvae exposed to DCR or arbutin at 72 hpf decreased in a dose-dependent manner and was lighter than that of the control larvae. The melanin contents of the zebrafish larvae treated with DCR extracts with concentrations of 10, 50, and 100 μg/ml were 92.95, 64.34, and 42.85%, respectively, of the melanin content of the control group, whereas those of the zebrafish larvae treated with the arbutin concentrations of 10, 50, and 100 μg/ml were 77.73, 53.47, and 29.63%, respectively, of the melanin content of the control group (Figure 2). Moreover, no abnormalities were observed in the morphologies of the DCR- and arbutin-treated zebrafish larvae. These results suggested that the DCR extract inhibited melanogenesis *in vivo*.

**FIGURE 3:**
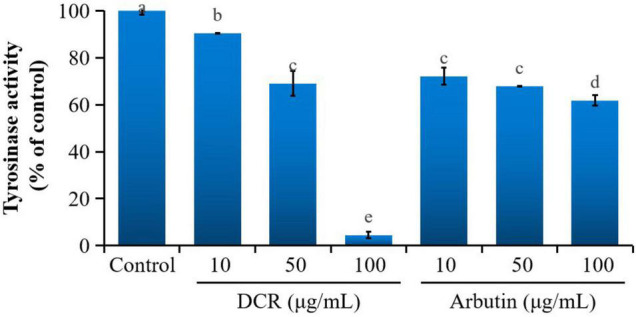
The effect of *Dryopteris crassirhizoma* rhizome (DCR) on the tyrosinase activity of zebrafish embryos. Different lower case letters indicate significant differences (*p* < 0.05).

To further determine whether melanogenesis inhibition in zebrafish embryos correlated with tyrosinase inhibition, the tyrosinase activity in zebrafish embryos was investigated. Treatment with DCR and arbutin considerably decreased the tyrosinase activity in zebrafish embryos at 72 hpf. The tyrosinase activities of the zebrafish embryos treated with DCR extracts at the concentrations of 10, 50, and 100 μg/ml were 90.34, 69.08, and 4.51%, respectively, of the tyrosinase activity of the control group, whereas those of the zebrafish treated with the arbutin concentrations of 10, 50, and 100 μg/ml were 72.14, 67.79, and 61.84%, respectively, of the tyrosinase activity of the control group (Figure 3). These results suggested that the DCR extract inhibited melanin synthesis *via* tyrosinase inhibition, thereby lowering pigmentation; moreover, these findings indicated that the DCR extract contained tyrosinase inhibitors.

### Characterization of the Tyrosinase Inhibitors in *Dryopteris crassirhizoma* Rhizome

**FIGURE 4:**
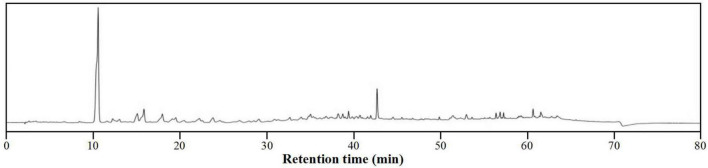
High-performance liquid chromatography (HPLC) profile of the *Dryopteris crassirhizoma* rhizome (DCR).

**FIGURE 5:**
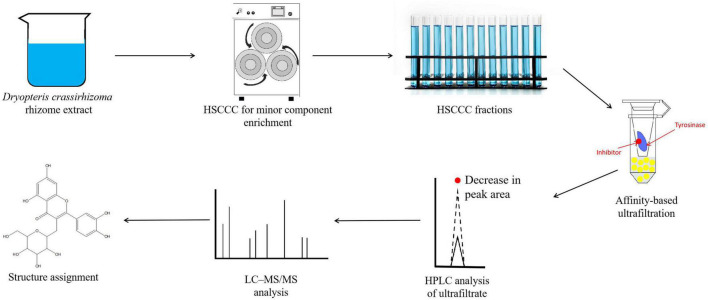
Schematic of the hyphenated method used to identify and characterize the tyrosinase inhibiting compounds in *Dryopteris crassirhizoma* rhizome (DCR). Here, HSCCC, LC–MS/MS, and HPLC denote high-speed counter-current chromatography, liquid chromatography–tandem mass spectrometry, and high-performance liquid chromatography, respectively.

To characterize the composition of the biologically active components of plant extracts, isolation is typically performed prior to evaluation or application. However, isolating biologically active components from DCR extracts is challenging because of the complex chemical composition of DCR (Figure 4). Alternatively, to characterize the tyrosinase inhibitors in DCR, an off-line hyphenation method combining HSCCC, affinity-based ultrafiltration, and LC–MS/MS was used in this study. A stepwise HSCCC was initially performed to enrich the minor components in DCR, and then, affinity-based ultrafiltration was conducted to identify potential tyrosinase inhibitors in DCR. Lastly, the structures of the potential tyrosinase inhibitors in DCR were determined using LC–MS/MS (Figure 5).

**FIGURE 6:**
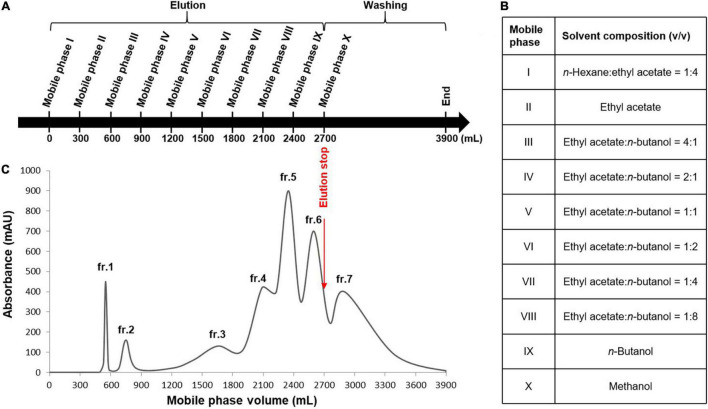
Stepwise high-speed counter-current chromatography (HSCCC) for the enrichment of the minor components in the *Dryopteris crassirhizoma* rhizome (DCR) extract. **(A)** HSCCC elution and washing steps. **(B)** Mobile phases of the solvent system. **(C)** HSCCC profile. Stationary phase: water–*n*-butanol [1:10 (v/v)], flow rate: 4 ml/min, rotation speed: 400 rpm, mode: tail-to-head.

**FIGURE 7:**
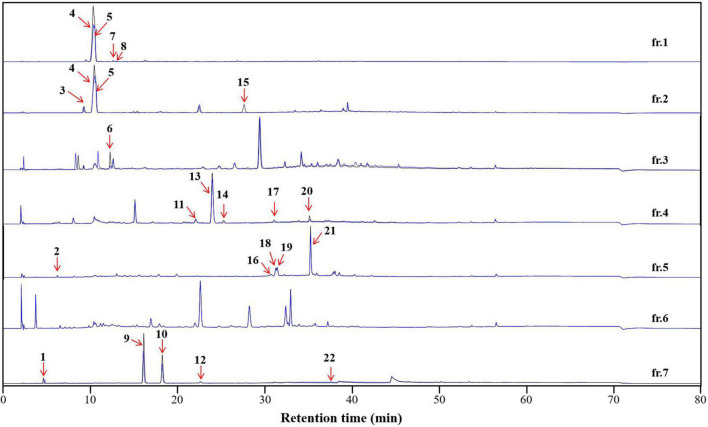
The affinity-based ultrafiltration chromatograms of the high-speed counter-current chromatography (HSCCC) fractions of the *Dryopteris crassirhizoma* rhizome (DCR) extract. The blue and black lines indicate fractions incubated with and without tyrosinase, respectively. The numbered peaks correspond to tyrosinase inhibitors.

An appropriate liquid phase comprising two immiscible liquids that satisfy the golden rules proposed by Ito is typically required for HSCCC separation (21). The HSCCC procedure was adopted because assessing the numerous solvent systems and comparing the partition coefficients of the components using HPLC or thin-layer chromatography is time-consuming. Moreover, the separation of compounds with similar partition coefficients is restricted by the low resolution of HSCCC. Stepwise HSCCC separation was performed to maximize the enrichment of the minor components in the primary components of the complex extracts and simplify solvent system selection. In this study, nine mobile phases of the n-hexane–EtOAc–n-BuOH–water solvent system with a wide polarity range were used without calculating the partition coefficients of the components (Figure 6). After mobile phase elution, the residual stationary phase in the coil was washed with methanol. Lastly, seven fractions were obtained, and their HPLC profiles are presented in Figure 7. The experimental data indicated that the primary components in DCR were collected in fractions 1 and 2, whereas the minor components were enriched in fractions 3–7.

**FIGURE 8:**
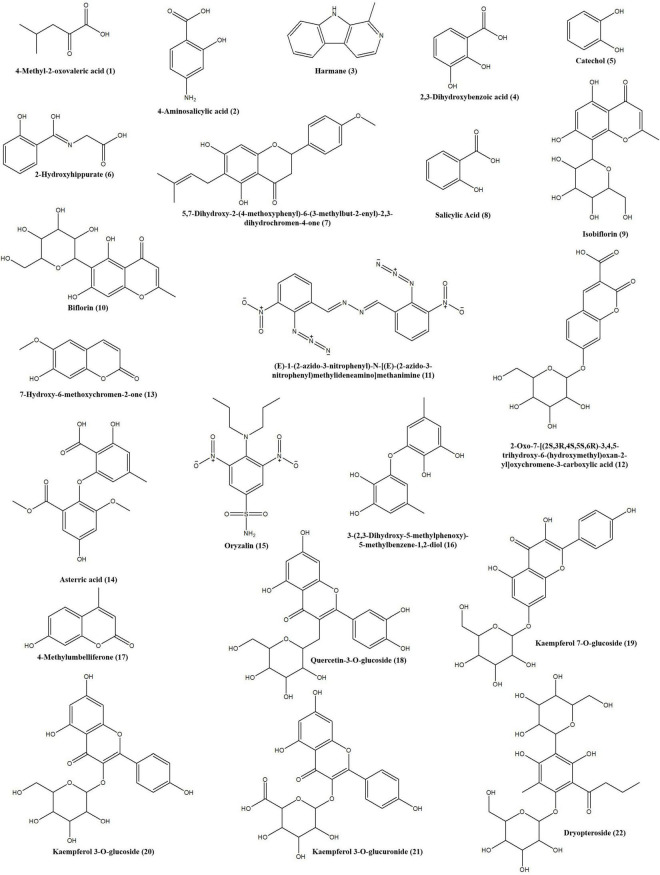
Structures of the tyrosinase inhibitors in the *Dryopteris crassirhizoma* rhizome (DCR) extract.

To characterize the tyrosinase inhibitors in DCR, the obtained HSCCC fractions were further analyzed *via* ultrafiltration combined with LC–MS/MS (22). The HPLC peak areas of the 22 compounds separated from DCR decreased, indicating their tyrosinase-inhibiting potentials. The structures of the 22 compounds, namely, 4-methyl-2-oxovaleric acid (**1**), 4-aminosalicylic acid (**2**), harmane (**3**), 2,3-dihydroxybenzoic acid (**4**), catechol (**5**), 2-hydroxyhippurate (**6**), 5,7-dihydroxy-2-(4-methoxyphenyl)-6-(3-methylbut-2-enyl)-2,3-dihydrochromen-4-one (**7**), salicylic acid (**8**), isobiflorin (**9**), biflorin, (**10**), (E)-1-(2-azido-3-nitrophenyl)-*N*-[(E)-(2-azido-3-nitrophenyl)methylideneamino]methanimine (**11**), 2-oxo-7-[(2S,3R,4S,5S,6R)-3,4,5-trihydroxy-6-(hydroxymethyl) oxan-2-yl]oxychromene-3-carboxylic acid (**12**), 7-hydroxy-6-methoxychromen-2-one (**13**), asterric acid (**14**), oryzalin (**15**), 3-(2,3-dihydroxy-5-methylphenoxy)-5-methylbenzene-1,2-diol (**16**), 4-methylumbelliferone (**17**), quercetin-3-O-glucoside (**18**), kaempferol 7-O-glucoside (**19**), kaempferol 3-O-glucoside (**20**), kaempferol 3-O-glucuronide (**21**), and dryopteroside (**22**), which are illustrated in Figure 7, were assigned by matching the precursors and MS/MS m/z values of the compounds with those compiled in libraries and comparing the data with references (Table 1 and Figure 8).

**TABLE 1:** Tyrosinase inhibitors in the *Dryopteris crassirhizoma* rhizome (DCR) extract identified using liquid chromatography–tandem mass (LC–MS/MS) in the negative ion mode.

No.	Retention time (min)	[M-H]^–^ (*m/z*)	MS/MS (*m/z*)	Formula	Mass error (mDa)	DBE^a^	Annotation
1	4.6	129.0548	114.9896; 111.0448	C_6_H_9_O_3_	−0.4	2.5	4-Methyl-2-oxovaleric
2	6.1	152.0348	134.0238; 109.0276	C_7_H_6_O_3_N	0.0	5.5	4-Aminosalicylic acid
3	9.2	181.0492	166.0517; 153.0260; 139.0379	C_12_H_9_N_2_	−27.4	9.5	Harmane
4	10.4	153.0186	109.0077	C_7_H_5_O_4_	−0.2	5.5	2,3-Dihydroxybenzoic acid
5	10.5	109.0288		C_6_H_5_O_2_	−0.2	4.5	Catechol
6	12.2	194.0252	152.0467; 137.0226; 108.0213	C_9_H_8_O_4_N	−20.1	6.5	2-Hydroxyhippurate
7	12.5	353.1316	165.0155; 139.0400; 121.0199	C_21_H_21_O_5_	−7.3	11.5	5,7-Dihydroxy-2-(4-methoxyphenyl)-6-(3-methylbut-2-enyl)-2,3-dihydrochromen-4-one
8	12.9	137.0236	121.0316; 108.0220	C_7_H_5_O_3_	−0.3	5.5	Salicylic acid
9	16.1	353.0865	233.0445; 205.0502	C_16_H_17_O_9_	−0.8	8.5	Isobiflorin
10	18.2	353.0866	233.0444; 205.0495	C_16_H_17_O_9_	−0.7	8.5	Biflorin
11	22.0	379.0689	335.0701; 162.0837	C_14_H_7_O_4_N_10_	3.7	16.5	(E)-1-(2-azido-3-nitrophenyl)-*N*-[(E)-(2-azido-3-nitrophenyl) methylideneamino]methanimine
12	22.6	367.0649	191.0335; 113.0234	C_16_H_15_O_10_	1.6	9.5	2-Oxo-7-[(2S,3R,4S,5S,6R)-3,4,5-trihydroxy-6-(hydroxymethyl)oxan-2-yl]oxychromene-3-carboxylic acid
13	24.0	191.0361	160.8393	C_10_H_6_O_4_	1.7	7.5	7-Hydroxy-6-methoxychromen-2-one
14	25.2	347.0768	303.0868; 259.0960	C_17_H_15_O_8_	0.1	10.5	Asterric acid
15	27.6	345.0525	285.0398; 219.0288; 191.0335; 177.0183; 149.0234; 125.0235; 109.0285	C_12_H_17_O_6_N_4_S	−34.4	6.5	Oryzalin
16	30.6	261.0757	233.0810; 204.0418	C_14_H_13_O_5_	−0.6	8.5	3-(2,3-Dihydroxy-5-methylphenoxy)-5-methylbenzene-1,2-diol
17	31.0	175.0366	129.0320	C_10_H_7_O_3_	−2.9	7.5	4-Methylumbelliferone
18	31.1	463.0878	287.0544; 113.0225	C_21_H_19_O_12_	0.1	12.5	Quercetin-3-*O*-glucoside
19	31.2	447.0935	327.0496	C_21_H_19_O_11_	0.8	12.5	Kaempferol 7-*O*-glucoside
20	35.1	447.0923	285.0391	C_21_H_19_O_11_	−0.4	12.5	Kaempferol 3-*O*-glucoside
21	35.1	461.0710	285.0395	C_21_H_17_O_12_	−1.0	13.5	Kaempferol 3-*O*-glucuronide
22	37.5	533.1864	41.31438; 251.0912	C_23_H_33_O_14_	−0.6	7.5	Dryopteroside

*^a^DBE, double bond equivalency.*

### Molecular Docking Prediction

The isolation of minor and trace compounds from complex extracts is laborious (23). Therefore, molecular docking analysis was performed to rapidly confirm the binding mechanisms of the identified compounds to tyrosinase, and the results are presented in Table 2 and Supplementary Figures 1–23. Each of the binuclear copper atoms of tyrosinase is bonded to three histidine residues and plays a critical role in catalytic reactions (24). We hypothesized that the interactions between **3**, **5**, **6**, **7**, and **8** and the amino acid residues of tyrosinase were hydrophobic, whereas the other 17 compounds interacted with tyrosinase *via* hydrogen bonding and hydrophobic interactions. In particular, **4**, **11**, **15**, and **16** formed hydrogen bonds with the amino acid residues of tyrosinase and did not interact with the copper atoms; **1**, **2**, **13**, **17**, and **21** formed hydrogen bonds with one copper atom and several amino acid residues; and **9**, **10**, **12**, **14**, **18**–**20**, and **22** formed hydrogen bonds with two copper atoms and several amino acid residues. The binding stabilities toward and affinities for tyrosinase of the 22 compounds were assessed using crash, polar, chem, G-, D-, potential mean force, and C-scores (Table 2). Specifically, the crash scores reflected the incorrect penetration of the ligand in the active site pocket of tyrosinase; the polar scores reflected the ligand region; the D-scores were calculated using the charges and van der Waals interactions between proteins and ligands; the potential mean force scores indicated the Helmholtz free energies for the protein–ligand atom interactions; the G-scores were derived by evaluating the internal energies of hydrogen-bonding, protein–ligand, and ligand–ligand interactions; and the chem scores described the points where hydrogen-bonding, lipophilic contact, and rotational entropy changed. Each scoring method was used for different purposes, and the individual scores could not comprehensively evaluate the ligand–tyrosinase interactions. Therefore, the C-scores were calculated by combining the crash, polar, chem, G-, D-, and potential mean force scores, and the results were used to comprehensively assess the ligand affinity (25). The C-scores decreased as follows: **22** (6.88) > **19** (6.79) > **10** (6.72) > **16** (6.54) > **6** (5.87) > **9** (5.05) > **21** (5.05) > **12** (4.89) > **14** (4.76) > **13** (4.72) > **1** (4.64) > **2** (4.51) > **20** (4.49) > **17** (4.46) > **11** (4.32) > **18** (4.19) > **5** (4.11) > **7** (4.08) > **15** (3.86) > **3** (3.82) > **8** (3.25) > **4** (3.06). Arbutin, which is a known tyrosinase inhibitor, forms hydrogen bonds with one of the copper atoms and the His61, His94, Glu256, Asn260, and His296 residues of tyrosinase, and its C-score was 4.83. These results indicate that the 22 compounds formed stable conformations in the active site pocket of tyrosinase. Among the 22 compounds, **8** and **21** have been reported as tyrosinase inhibitors (26, 27), whereas **4** and **5** have been reported as tyrosinase substrates (28, 29). The rest of the 22 compounds have not been reported to interact with tyrosinase. Kubo et al. (30) reported that **18** and **20** were not tyrosinase inhibitors or substrates. However, Ohguchi et al. (31) demonstrated that **18** considerably inhibited the production of melanin in mouse B16 melanoma cells by suppressing tyrosinase expression. The inhibitory effects of the 22 compounds will be investigated in a future study.

**TABLE 2:** Docking scores and predicted interactions between the 22 *Dryopteris crassirhizoma* rhizome (DCR) compounds with tyrosinase inhibitory activity and tyrosinase.

No.	Compounds	Hydrogen bonds	Hydrophobic interactions	Crash score	Polar score	D-score	PMF score	G-score	Chem score	C-score
1	4-Methyl-2-oxovaleric	Copper, His61, His85	Glu256, His259, Asn260, His263, Ala286	–1.04	1.85	–76.68	–71.85	–111.67	–14.85	4.64
2	4-Aminosalicylic acid	Copper, His61, Asn260	His85, His259, His263, Met280, Gly281, Ala286, Phe292	–1.52	2.89	–88.74	–76.79	–133.92	–22.01	4.51
3	Harmane	−	His61, His85, His244, Glu256, His259, Asn260, His263, Met280, Ser282, Val283, Ala286	–1.29	0	–114.57	–66.35	–174.07	–21.81	3.82
4	2,3-Dihydroxybenzoic acid	Met280	His61, His85, His259, Asn260, His263, Phe264, Gly281, Ser282, Val283, Ala286	–0.54	0.81	–90.81	–53.50	–127.26	–12.80	3.06
5	Catechol	−	Gly281, Phe264, Met280, His259, His263, Asn260	–0.36	2.12	–66.08	–21.31	–105.08	–16.52	4.11
6	2-Hydroxyhippurate	−	His259, Asn260, His263, Phe264, Met280, Gly281, Ser282, Val283, Ala286	–0.48	2.15	–97.06	–24.56	–106.02	–14.72	5.87
7	5,7-Dihydroxy-2-(4-methoxyphenyl)-6-(3-methylbut-2-enyl)-2,3-dihydrochromen-4-one	−	His61, His85, His259, Asn260, His263, Phe264, Gly281, Ser282, Val283, Ala286	–1.11	0.33	–132.01	–44.81	–164.31	–17.90	4.08
8	Salicylic acid	−	His61, His85, His259, Asn260, His263, Phe264, Met280, Gly281, Ser282, Val283, Als286,	–0.51	0.79	–76.61	–39.99	–116.19	–13.76	3.25
9	Isobiflorin	Coppers, Met280, Gly281	His61, His85, Phe90, His244, Glu256, His259, Asn260, His263, Phe264, Ser282, Val283, Ala286, Phe292	–3.92	4.18	–189.90	–105.66	–230.55	–28.35	5.05
10	Biflorin	Coppers, His259, His263, Met280	His61, His85, His244, Val248, Asn260, Phe264, Gly281, Ser282, Val283, Ala286	–2.23	3.94	–176.80	–102.67	–177.34	–20.85	6.72
11	(E)-1-(2-azido-3-nitrophenyl)-*N*-[(E)-(2-azido-3-nitrophenyl)methylidene amino]methanimine	Als246, Met280	His85, Gly86, His244, Val248, Asn260, His263, Phe264, Val283, Ala286, Glu322	–0.83	2.08	–108.83	–81.52	–164.28	–19.91	4.32
12	2-Oxo-7-[(2S,3R,4S,5S,6R)-3,4,5-trihydroxy-6-(hydroxymethyl)oxan-2-yl]oxychromene-3-carboxylic acid	Coppers, His61, His85	Phe90, Val248, His259, Asn260, His263, Phe264, Met280, Gly281, Ser282, Val283, Ala286	–2.99	2.43	–152.21	–120.32	–186.86	–17.16	4.89
13	7-Hydroxy-6-methoxychromen-2-one	Copper, His61, Asn260	His85, His244, Glu256, His259, His263, Val283, Ala286, Phe292	–0.75	2.97	–118.80	–103.76	–115.82	–23.66	4.72
14	Asterric acid	Coppers, Glu256, His259, His263	His61, His85, Phe90, His244, Val248, Asn260, Phe264, Met280, Gly281, Ser282, Val283	–4.09	3.32	–169.85	–104.25	–209.54	–24.69	4.76
15	Oryzalin	Gly281	His85, Asn260, His263, Phe264, Met280, Ser282, Val283	–3.59	1.66	–135.64	–34.37	–209.64	–20.05	3.86
16	3-(2,3-Dihydroxy-5-methylphenoxy)-5-methylbenzene-1,2-diol	His244	His61, His85, His259, Asn260, His263, Phe264, Met280, Gly281, Als286,	–1.23	3.05	–138.85	–58.11	–172.59	–22.26	6.54
17	4-Methylumbelliferone	Copper, His61	His85, Glu256, His259, Asn260, His263, Phe264, Met280, Gly281, Val283, Phe292	–0.88	1.89	–109.82	–77.08	–130.26	–23.36	4.46
18	Quercetin-3-*O*-glucoside	Coppers, His61, His85, His94, Met280	His244, Val248, His259, Asn260, His263, Phe264, Arg268, Pro277, Gly281, Ser282, Val283, Ala286, His296	–3.00	2.46	–170.51	–121.99	–215.29	–24.17	4.19
19	Kaempferol 7-*O*-glucoside	Coppers, His244, His259, His263, Met280,	His85, Asn260, Phe264, Gly281, Ser282, Val283, Pro284, Ala286	–1.51	3.97	–166.57	–121.68	–106.86	–20.38	6.79
20	Kaempferol 3-*O*-glucoside	Coppers, His61, His85, Arg268, Met280, Ser282, Val283	Val248, His259, Asn260, His263, Phe264, Gly281, Pro284, Ala286	–3.45	3.93	–163.03	–108.03	–131.90	–23.34	4.49
21	Kaempferol 3-*O*-glucuronide	Copper, His61, His85, Arg268, Met280	Val248, His259, Asn260, His263, Phe264, Pro277, Gly281, Ser282, Val283, Ala286	–2.50	3.04	–169.61	–120.77	–152.54	–23.33	5.05
22	Dryopteroside	Coppers, His61, His85, Met280, Ser282, Val283	His244, Val248, His259, Asn260, His263, Phe264, Gly281, Pro284, Ala286	–3.32	3.56	–211.41	–116.56	–216.75	–14.49	6.88
	Arbutin	Copper, His61, His94, Glu256, Asn260, His296	His85, His244, Val248, His259, His263, Phe264, Val283, Ala286, Phe292	–2.02	3.35	–148.86	–109.69	–175.31	–18.62	4.83

## Conclusion

In this study, the inhibitory activity of DCR on mushroom tyrosinase was assessed, and the effect of DCR on melanogenesis in zebrafish embryos was evaluated. Our results demonstrated that DCR could serve as an effective tyrosinase inhibitor and significantly decreased the melanin content and tyrosinase activity in zebrafish embryos. An offline hyphenated method comprising HSCCC, affinity-based ultrafiltration, and LC–MS/MS was used to identify and characterize the 22 DCR compounds with tyrosinase inhibitory activity. Lastly, *in silico* molecular docking was performed to rapidly evaluate the binding mechanisms of the 22 compounds on tyrosinase, and our results indicated that the compounds formed stable conformations in the active site pocket of tyrosinase.

## Data Availability Statement

The original contributions presented in the study are included in the article/Supplementary Material, further inquiries can be directed to the corresponding author.

## Ethics Statement

All animal experiments were performed in accordance with the guidelines and approval of the Institutional Animal Care and Use Committee (IACUC) of Hebei University (IACUC-20180051). Written informed consent was obtained from the owners for the participation of their animals in this study.

## Author Contributions

ZW: conceptualization, writing the original draft preparation, project administration, and funding acquisition. HY: conceptualization, writing, reviewing, editing, and funding acquisition. NW: methodology and investigation. DH: writing, reviewing, and editing. All authors contributed to the article and approved the submitted version.

## Conflict of Interest

The authors declare that the research was conducted in the absence of any commercial or financial relationships that could be construed as a potential conflict of interest.

## Publisher’s Note

All claims expressed in this article are solely those of the authors and do not necessarily represent those of their affiliated organizations, or those of the publisher, the editors and the reviewers. Any product that may be evaluated in this article, or claim that may be made by its manufacturer, is not guaranteed or endorsed by the publisher.

## Abbreviations

DCR: *Dryopteris crassirhizoma* rhizome
EtOAc: ethyl acetate
hpf: hours post-fertilization
HPLC: high-performance liquid chromatography
HSCCC: high-speed countercurrent chromatography
LC–MS/MS: liquid chromatography–tandem mass spectrometry
*n*-BuOH: *n*-butanol
PBS: phosphate-buffered saline
UV: ultraviolet.
