# Supplementary material for: Characterization of Tyrosinase Inhibitors in Dryopteris crassirhizoma Rhizome Using a Combination of High-Speed Counter-Current Chromatography, Affinity-Based Ultrafiltration, and Liquid Chromatography–Tandem Mass Spectrometry
Source: Front Nutr. 2022 Apr 18;9:862773. doi: 10.3389/fnut.2022.862773 (PMC9063005; doi:10.3389/fnut.2022.862773)
Supplement: Supplementary file 1 [file Data_Sheet_1.DOCX]

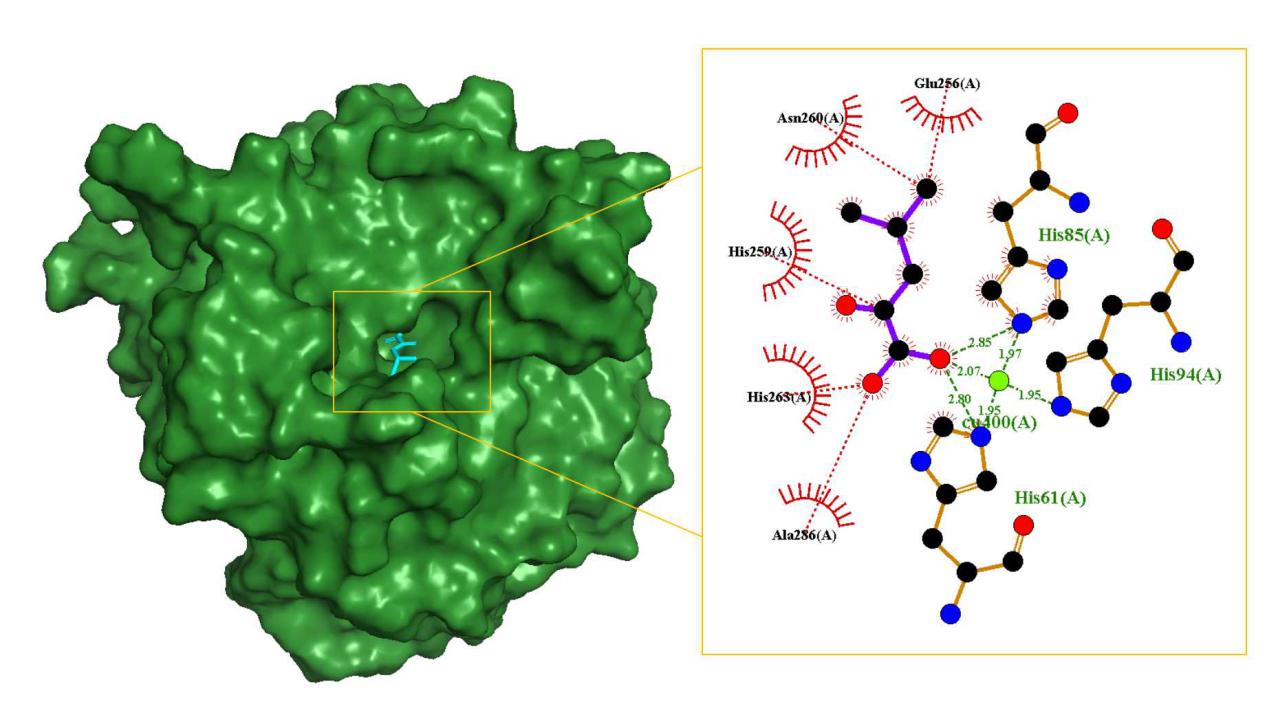
Figure S1. Tyrosinase docked with 4-methyl-2-oxovaleric.


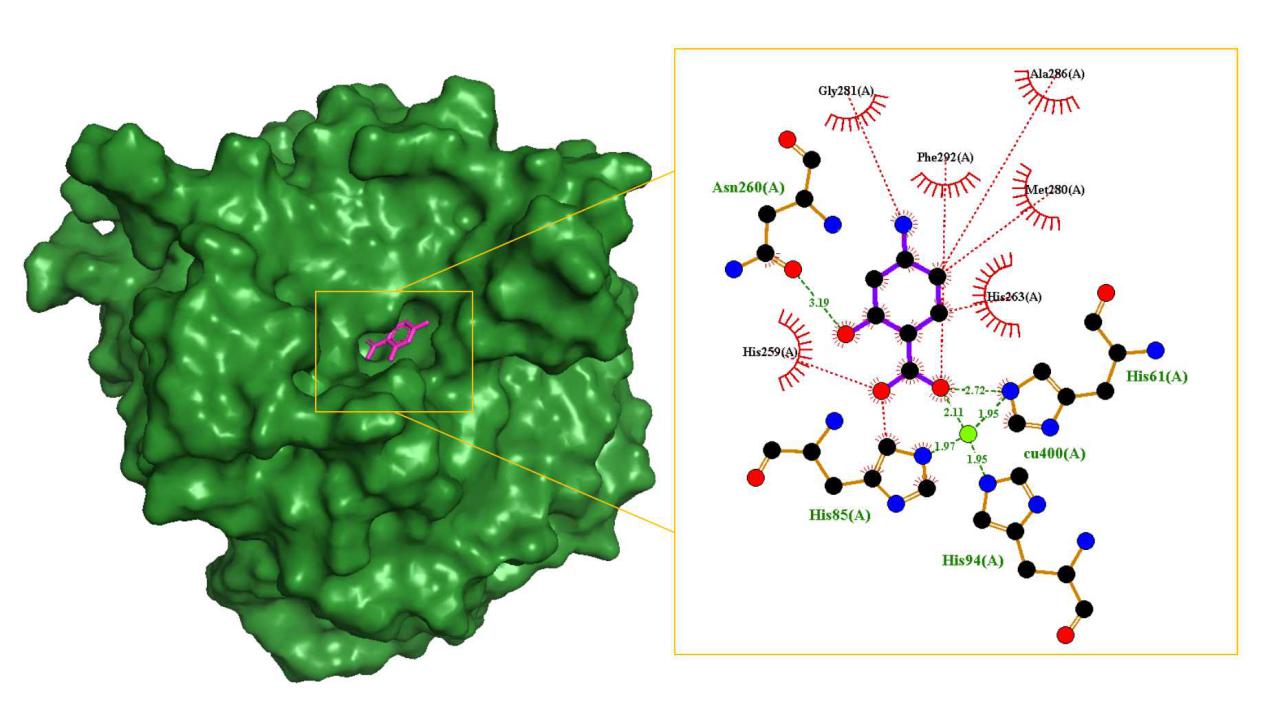


Figure S2. Tyrosinase docked with 4-aminosalicylic acid.

.


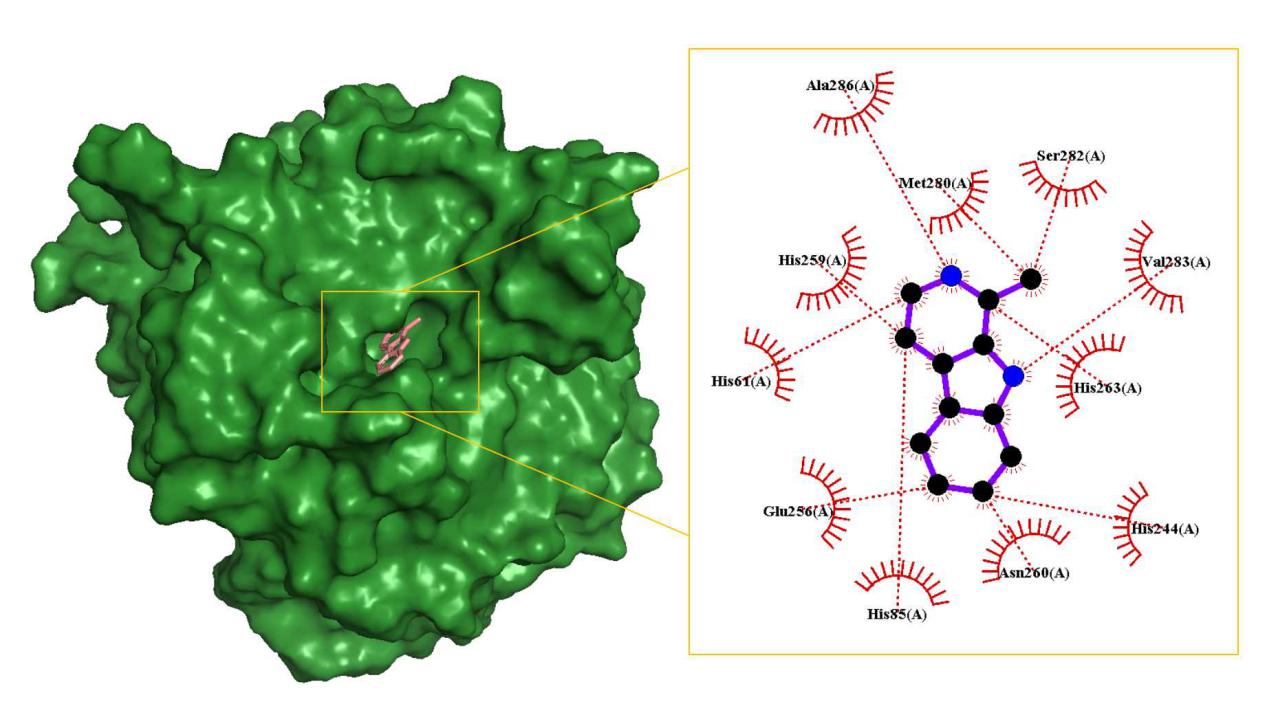


Figure S3. Tyrosinase docked with harmane.


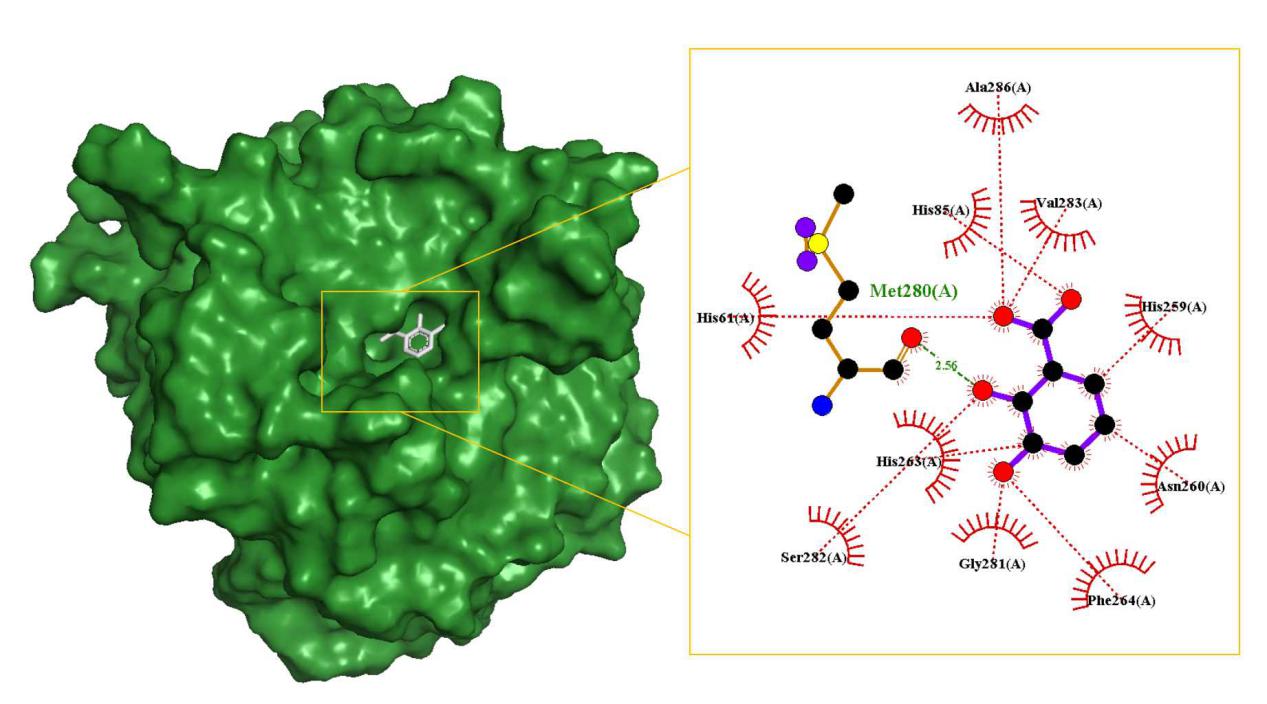


Figure S4. Tyrosinase docked with 2,3-dihydroxybenzoic acid.


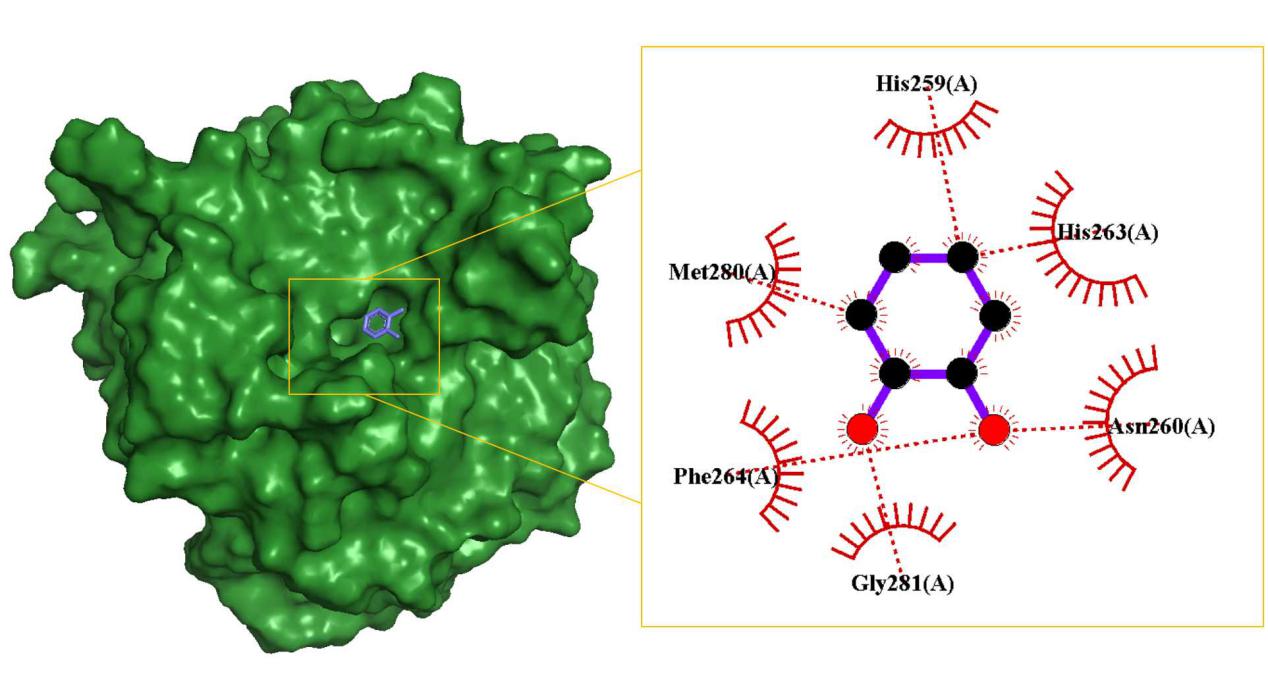


Figure S5. Tyrosinase docked with catechol.


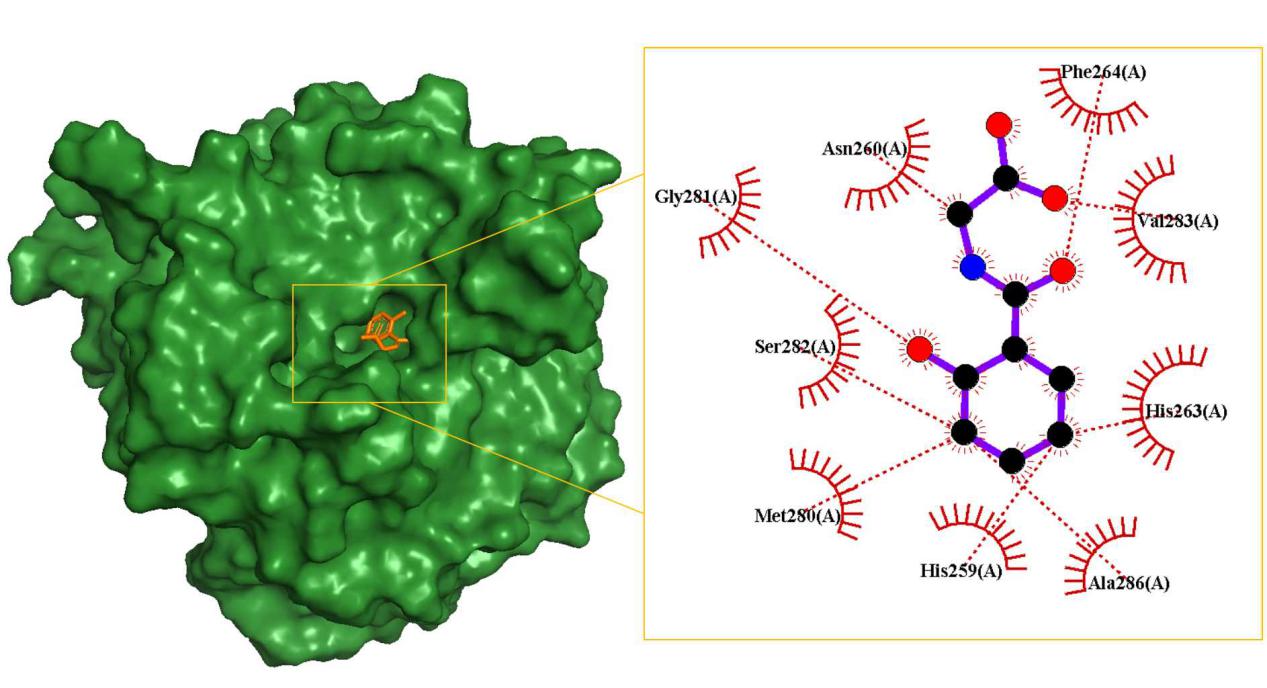


Figure S6. Tyrosinase docked with 2-hydroxyhippurate.


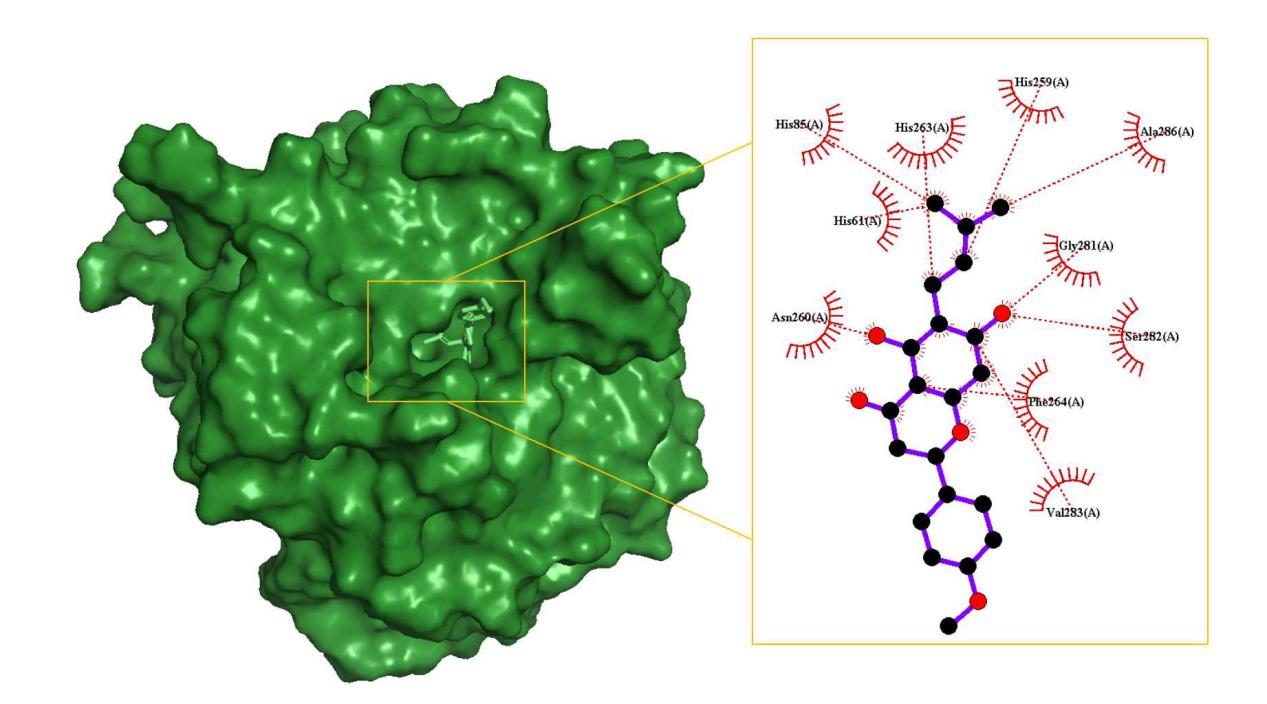


Figure S7. Tyrosinase docked with 5,7-dihydroxy-2-(4-methoxyphenyl)-6-(3-methylbut-2-enyl)-2,3-dihydrochromen-4-one.


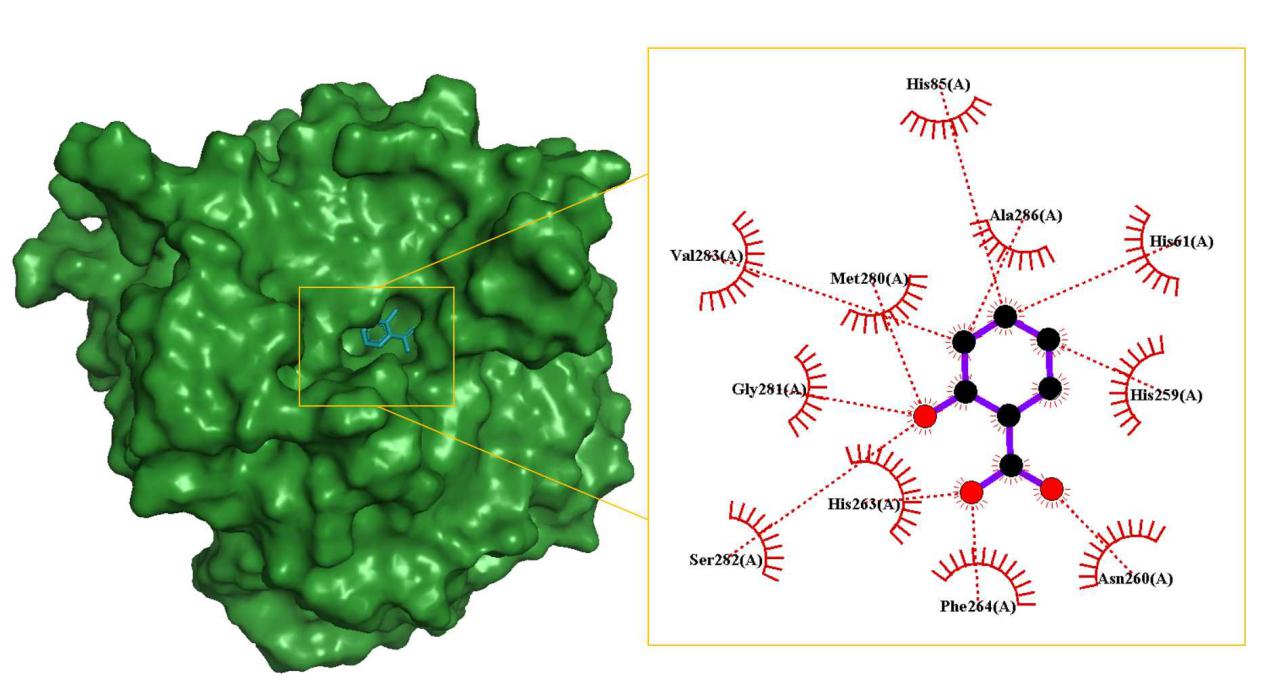


Figure S8. Tyrosinase docked with salicylic acid.


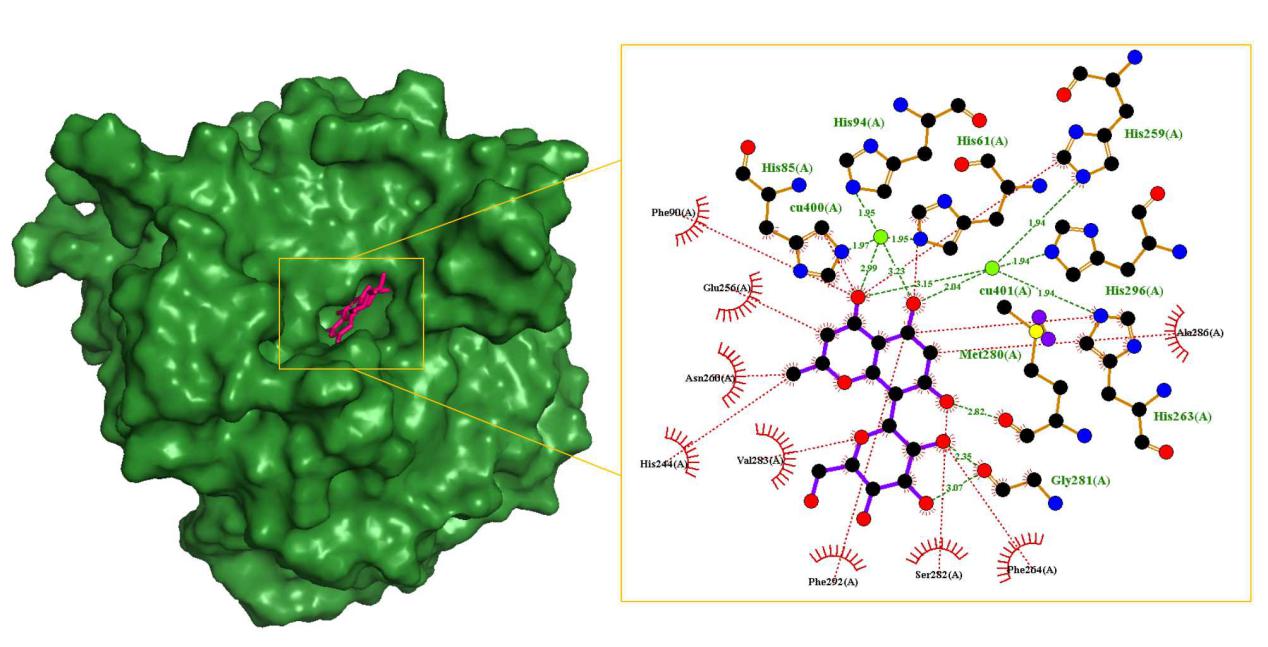


Figure S9. Tyrosinase docked with isobiflorin.


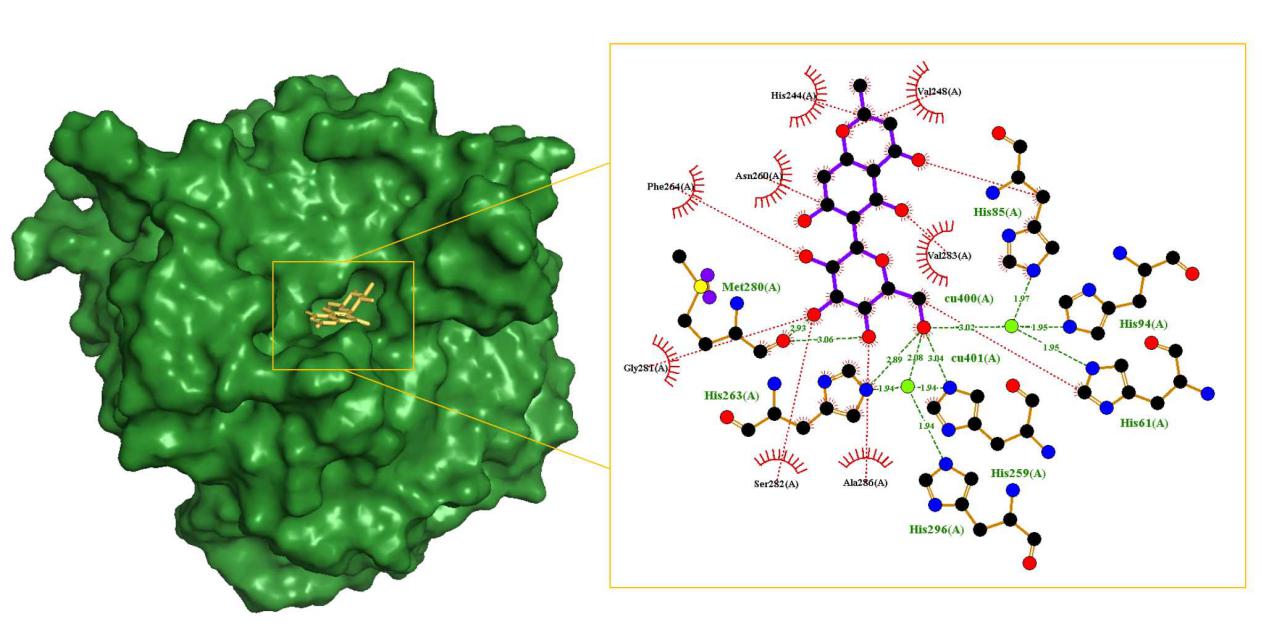


Figure S10. Tyrosinase docked with biflorin.


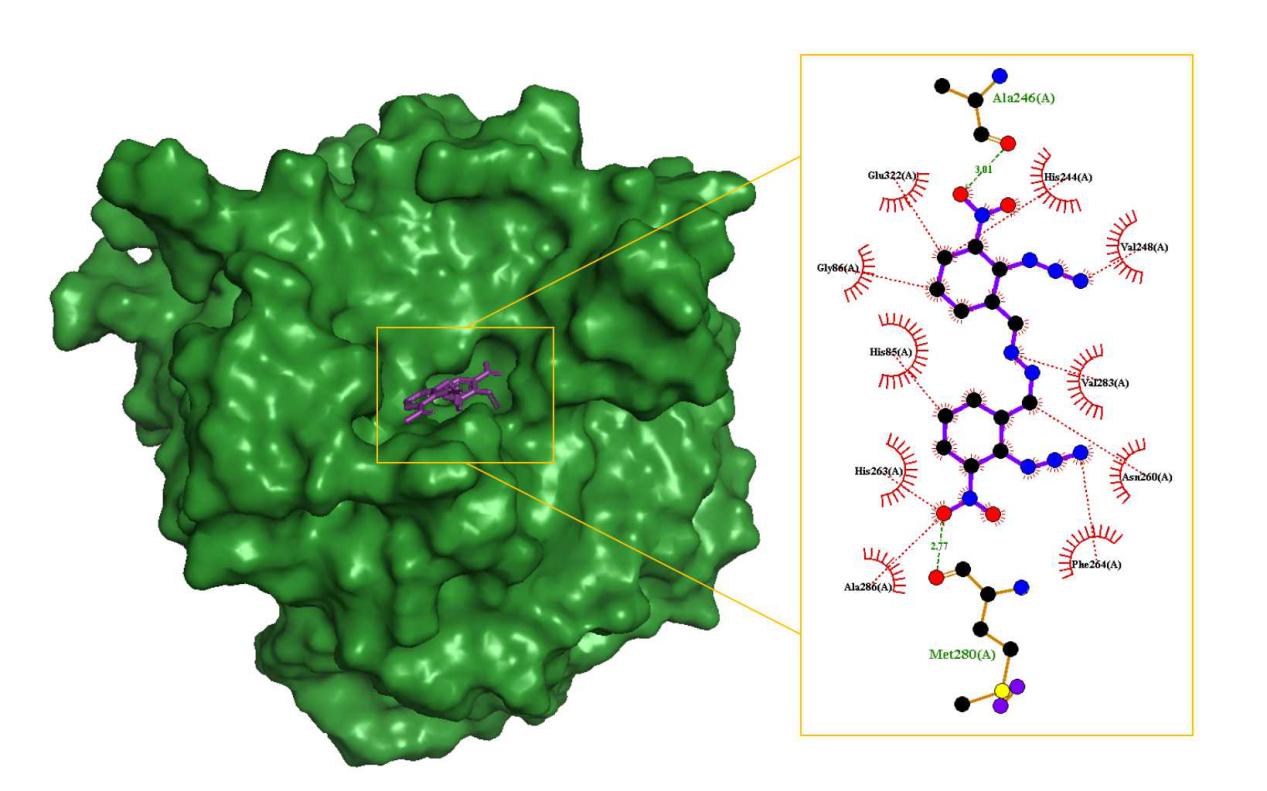


Figure S11. Tyrosinase docked with (E)-1-(2-azido-3-nitrophenyl)-N-[(E)-(2-azido-3-nitrophenyl)methylideneamino]methanimine.


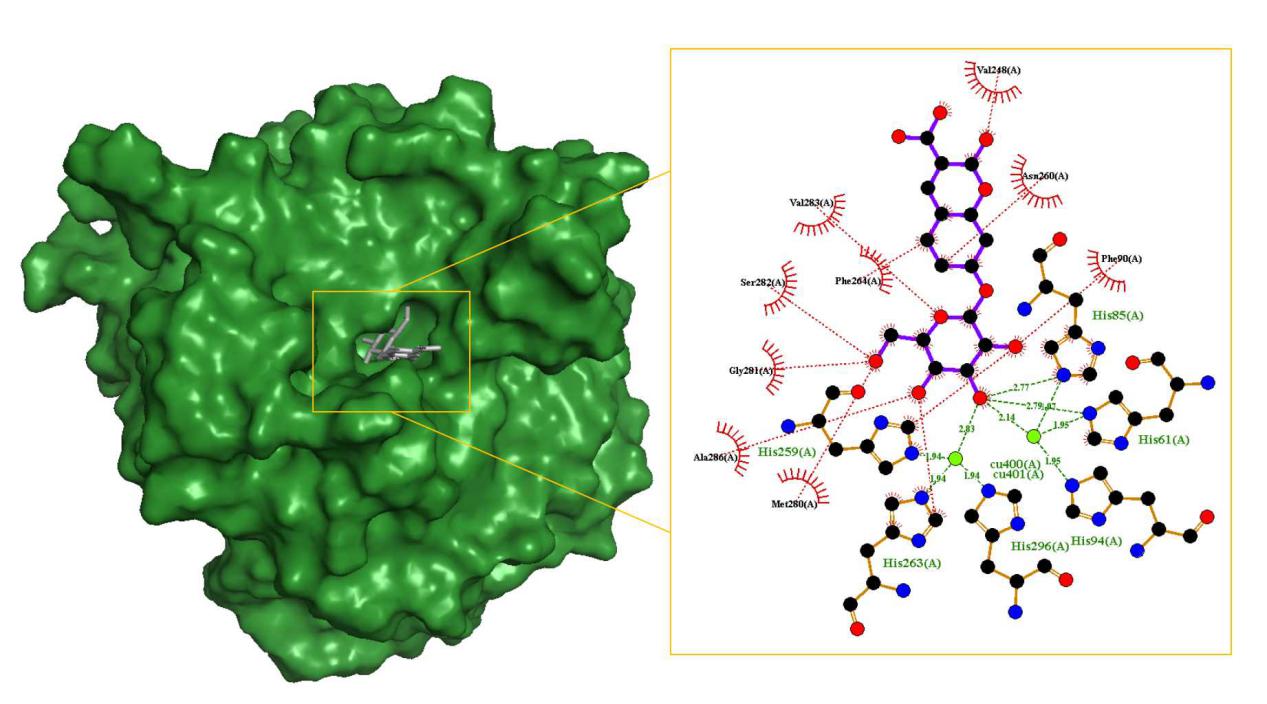


Figure S12. Tyrosinase docked with 2-oxo-7-[(2S,3R,4S,5S,6R)-3,4,5-trihydroxy-6-(hydroxymethyl)oxan-2-yl]oxychromene-3-carboxylic acid.


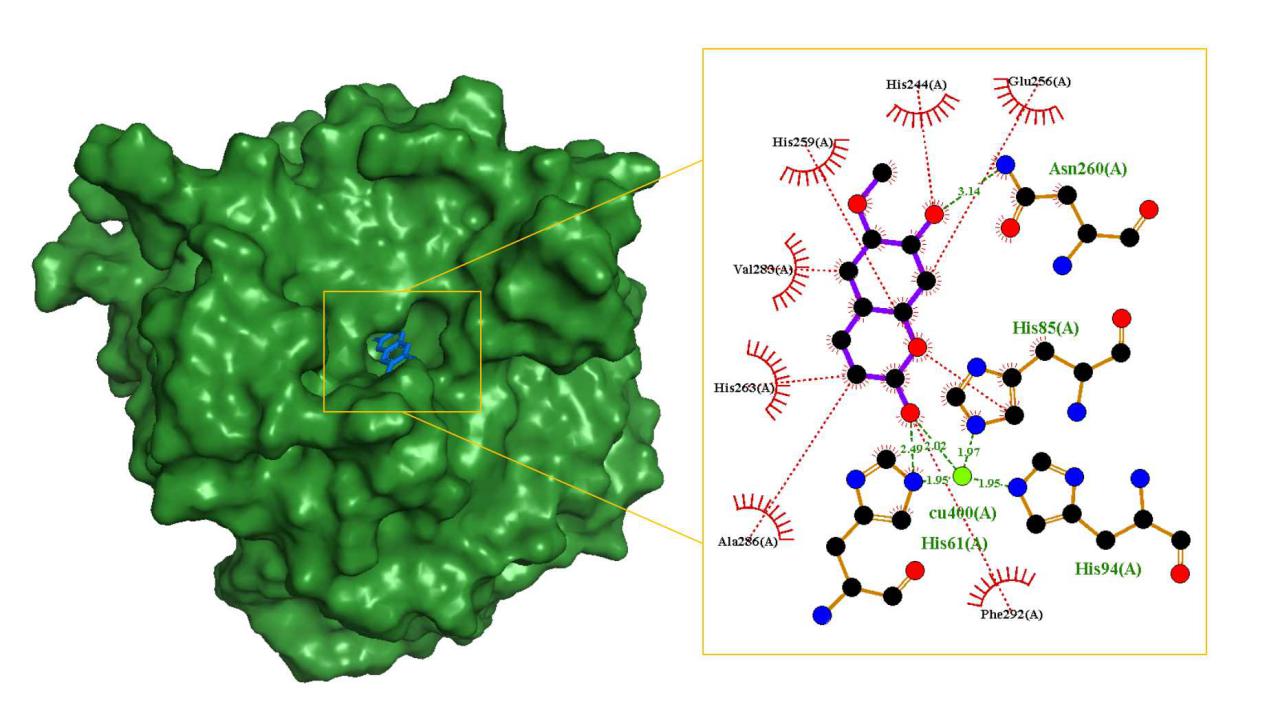


Figure S13. Tyrosinase docked with 7-hydroxy-6-methoxychromen-2-one.


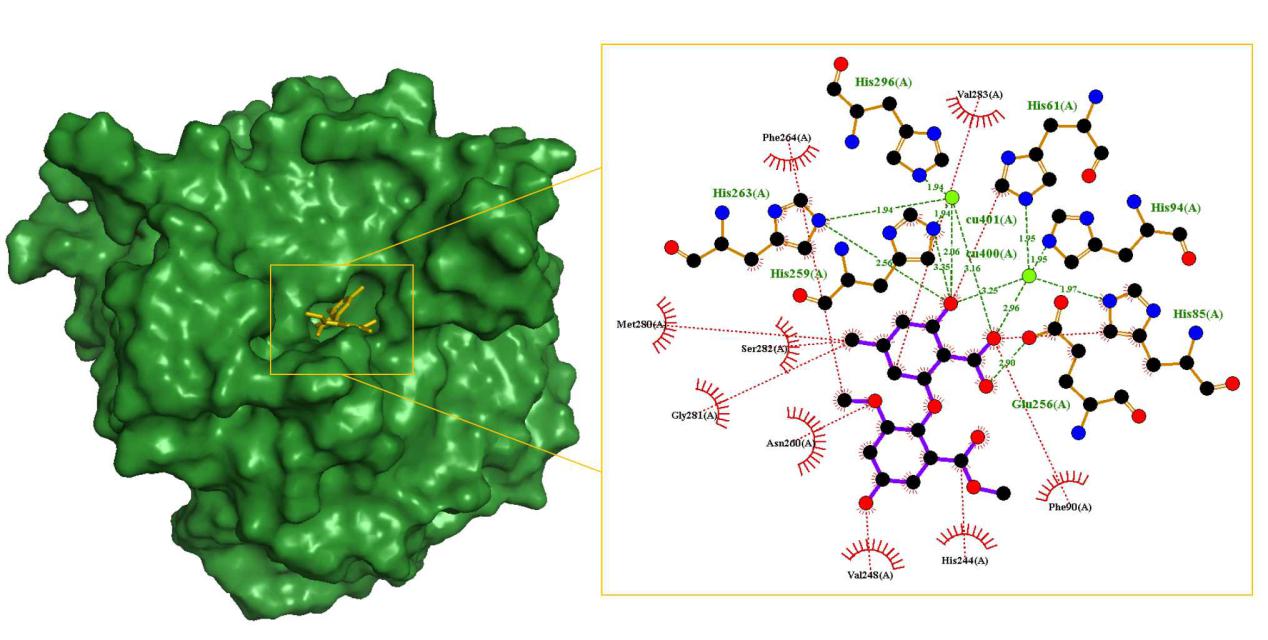


Figure S14. Tyrosinase docked with asterric acid.


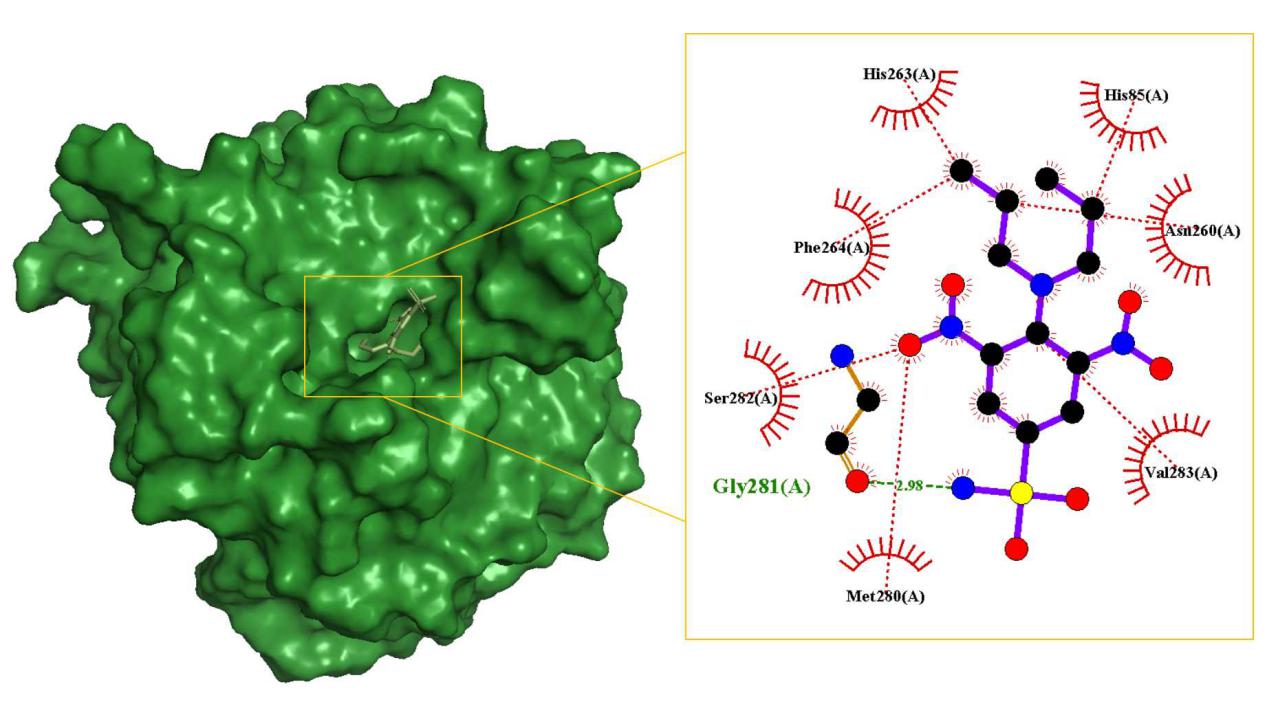


Figure S15. Tyrosinase docked with oryzalin.


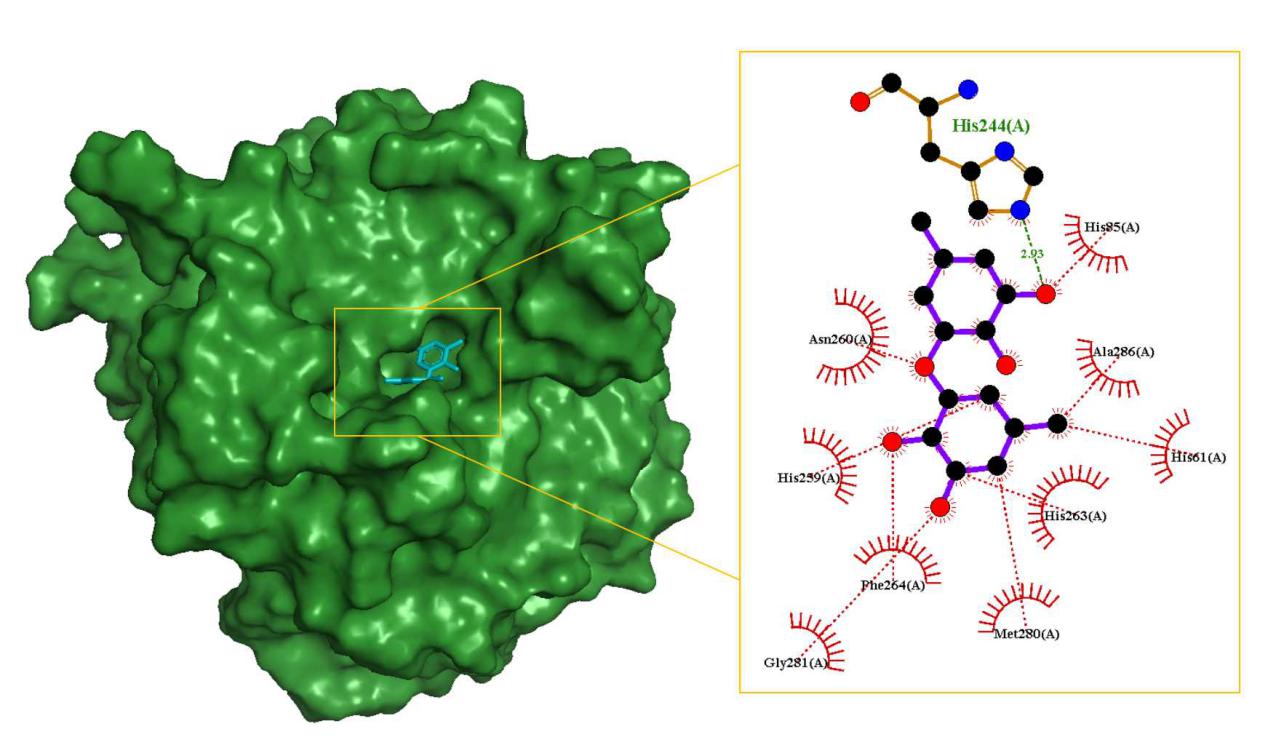


Figure S16. Tyrosinase docked with 3-(2,3-dihydroxy-5-methylphenoxy)-5-methylbenzene-1,2-diol.


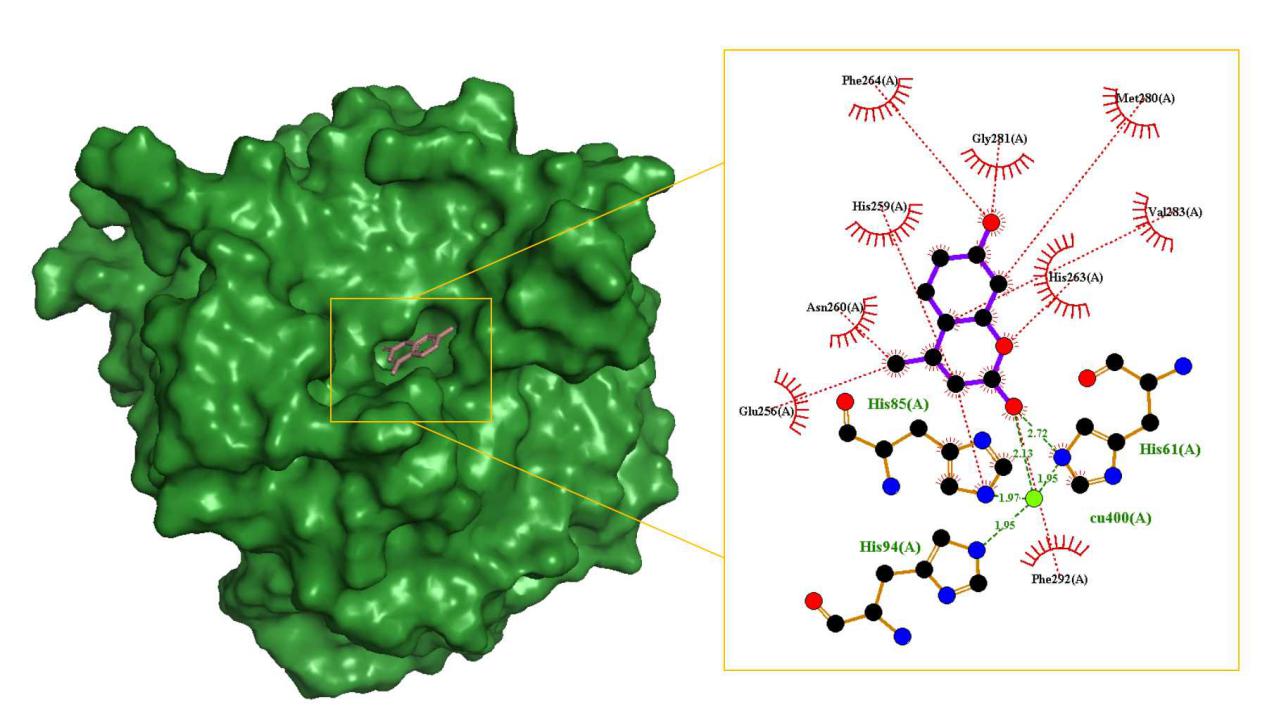


Figure S17. Tyrosinase docked with 4-methylumbelliferone.


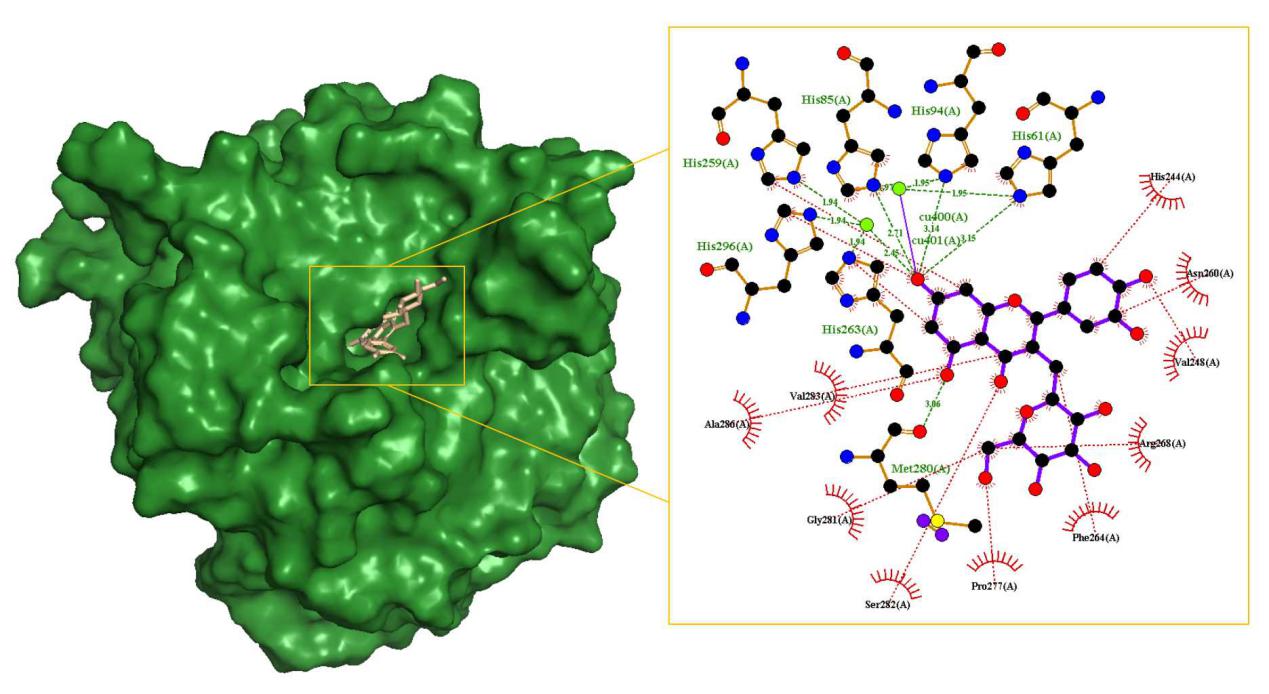


Figure S18. Tyrosinase docked with quercetin-3-O-glucoside.


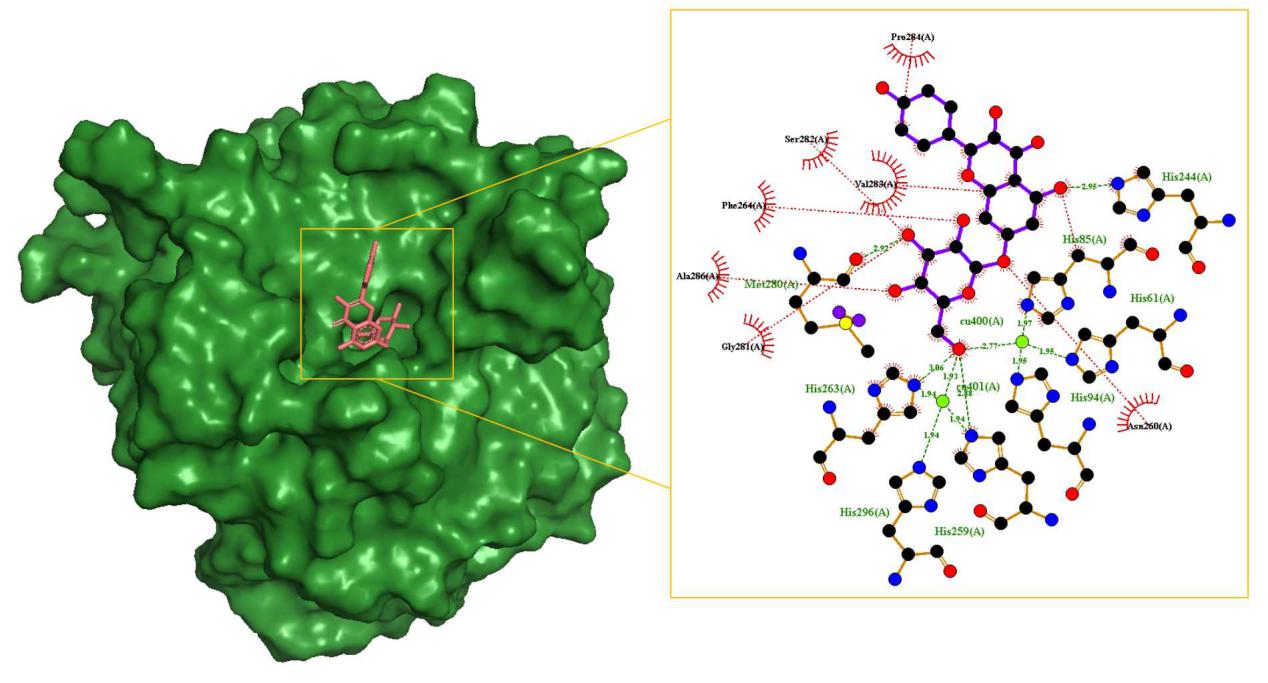


Figure S19. Tyrosinase docked with kaempferol 7-O-glucoside.


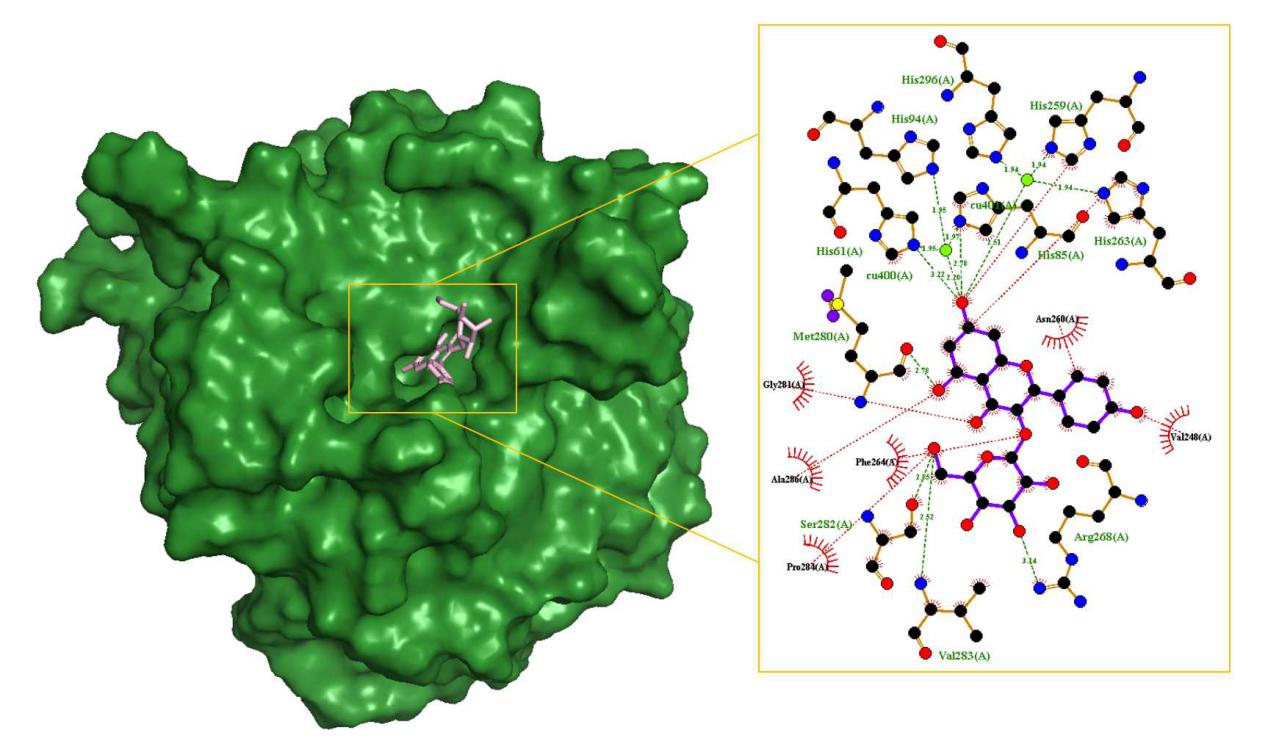


Figure S20. Tyrosinase docked with kaempferol 3-O-glucoside.


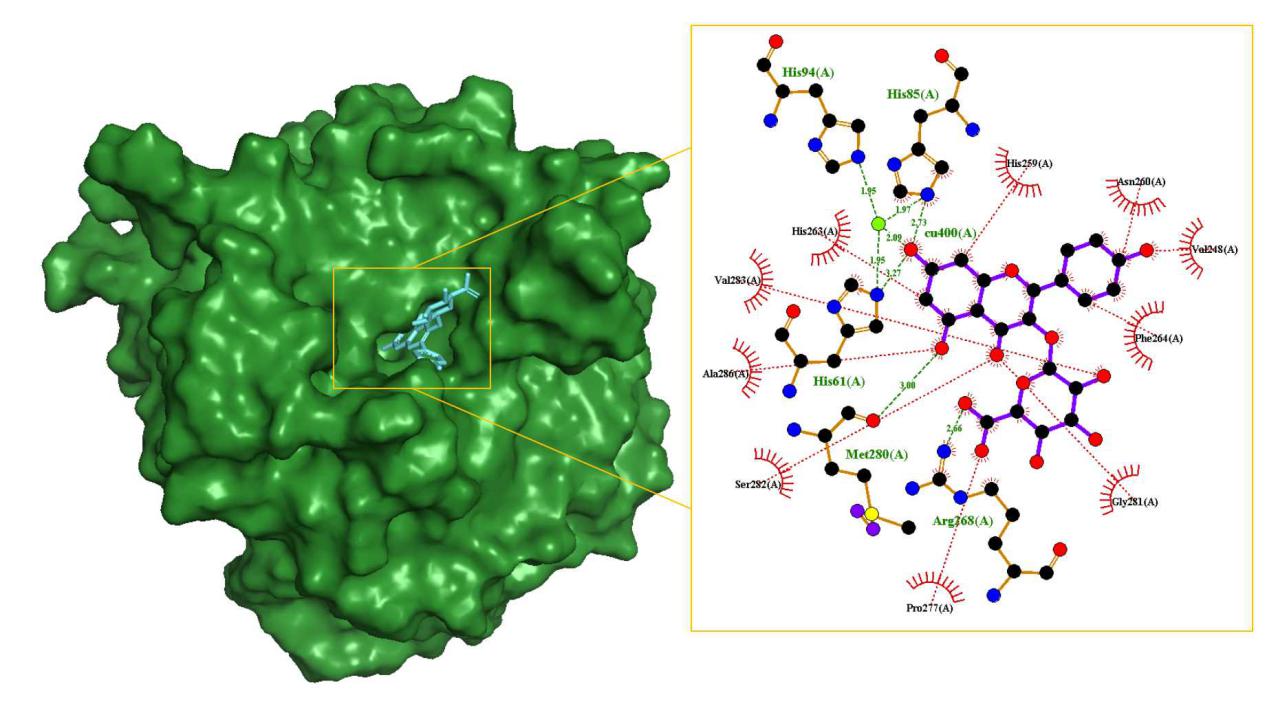


Figure S21. Tyrosinase docked with kaempferol 3-O-glucuronide.


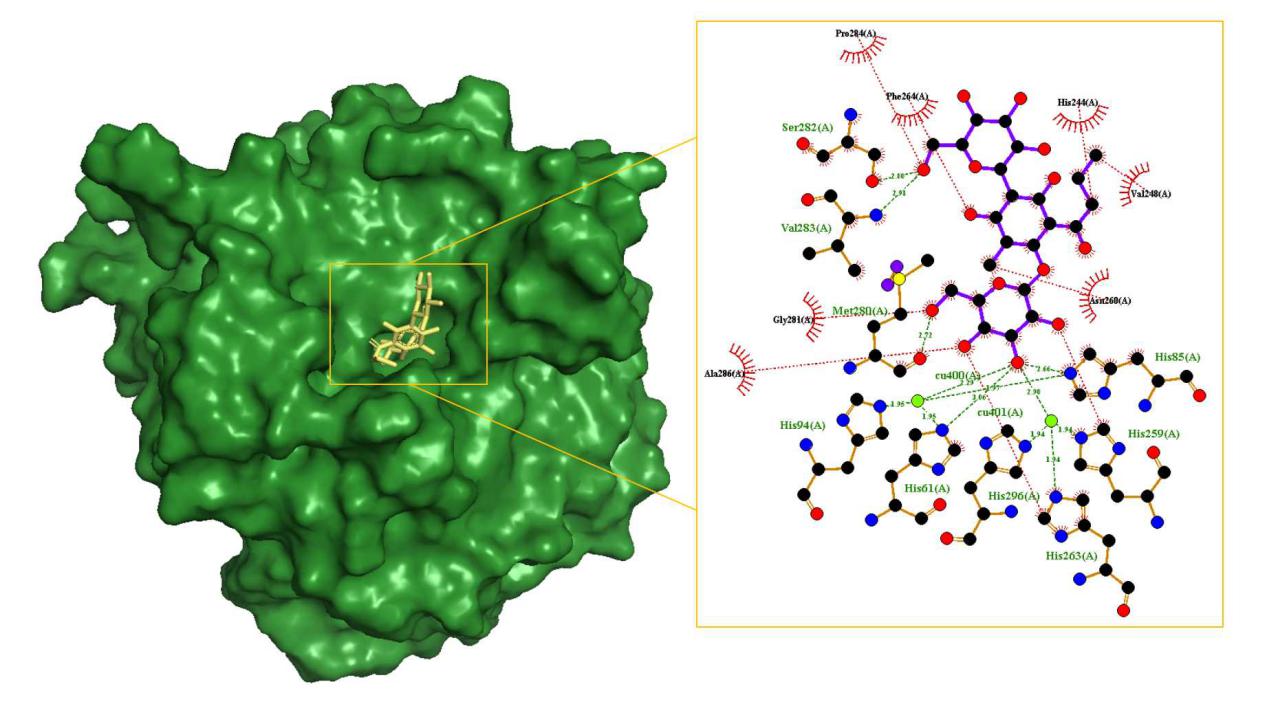


Figure S22. Tyrosinase docked with dryopteroside.


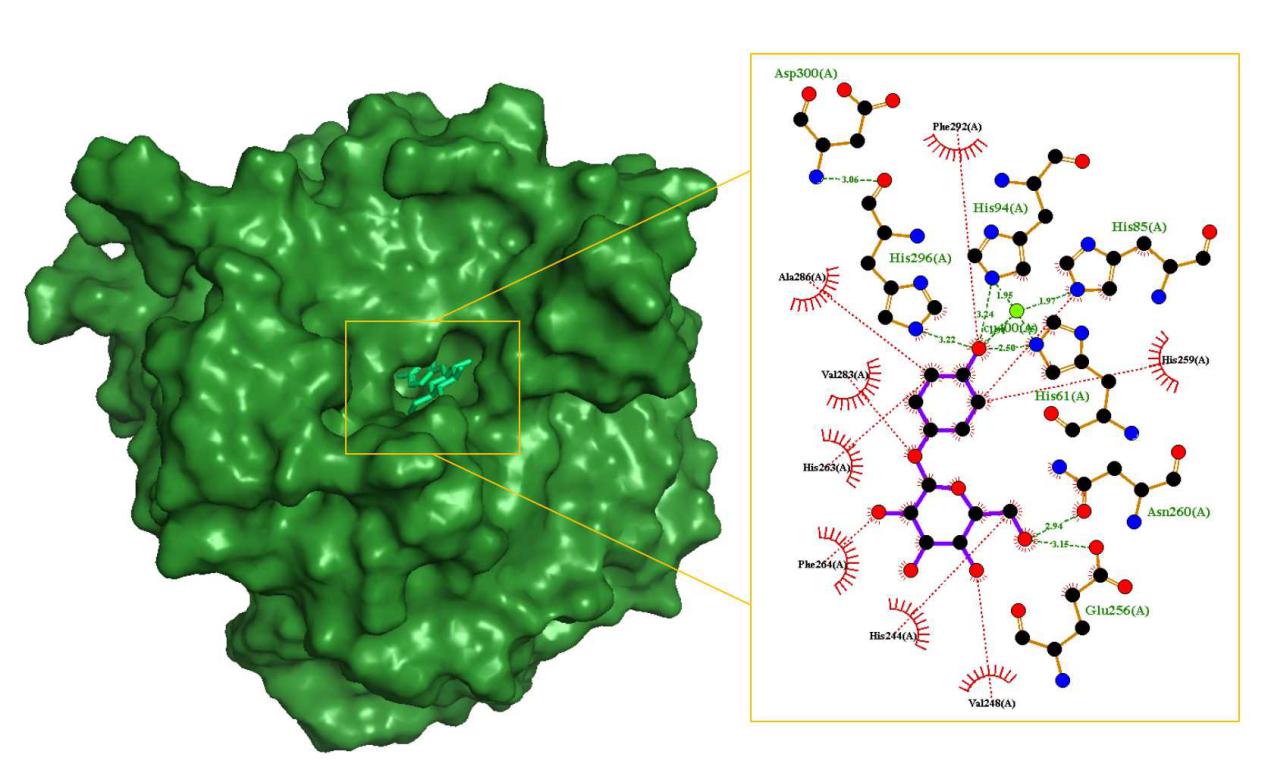


Figure S23. Tyrosinase docked with arbutin.
